# Protective effect of chaige anti-alcoholic granules on acute alcoholic liver injury in rats and acute toxicity in mice

**DOI:** 10.3389/fphar.2025.1595544

**Published:** 2025-08-01

**Authors:** Ying Chen, Feiyu Zhao, Xing Sang, Rongzhen Zhang, Mengfei Bi, Meimei Tang, RongBin Wang, Hongting Wang, Cunqin Wang

**Affiliations:** ^1^ School of Pharmacy, Wannan Medical College, Wuhu, China; ^2^ School of Pharmacy, Shanghai University of Traditional Chinese Medicine, Shanghai, China; ^3^ Department of Pharmacy, Xuyi People’s Hospital, Huai’an, Jiangsu, China; ^4^ Department of Emergency Medicine, Wuhu Hospital of Traditional Chinese Medicine, Wuhu, China; ^5^ Institute of Chinese Medicine Resources, Anhui College of Traditional Chinese Medicine, Wuhu, China

**Keywords:** alcohol, hepatoprotection, oxidative stress, inflammation, cag

## Abstract

**Background and aim:**

Alcoholic liver disease (ALD), caused by consumption of alcohol, with high morbidity and mortality, whose effective interventions is essential. Chinese medicine has a long history of detoxification, and Chaige anti-alcoholic granules (CAG) is an accepted formula including 13 Chinese herbs with definite detoxifying and liver-protecting effects in clinical for acute alcohol intoxication. However, the underlying mechanism is unclear.

**Methods:**

In this study, mice were selected for acute toxicity experiments with the increasing drug concentration to 0.78 mg⋅mL-1. Body weight changes, organ indices, liver and kidney histological observations were performed after 2 weeks. ALT, AST, BUN, and creatinine in mice serum were detected by the kits. A rat model of alcoholic liver injury (ALI) was established by gavage of Chinese wine with 56% alcohol, which was intragastrically received with CAG at 1575, 3150 and 6300 mg⋅kg⋅day-1 for 2 weeks, respectively, while positive group 100 mg⋅kg⋅day-1 metadoxine. The organ indices were measured, and the protective effect of CAG on ALI was determined using kits, ELISA, histopathology, and western blotting.

**Results:**

The results of the acute toxicity experiment showed that the mice were alive normally and the organ index, liver and kidney histopathology, and serum biochemical indicators showed no significant difference between the control group and the CAG-treated groups. The results of hepatoprotective effect of CAG in rat showed that compared with the control group, the liver index, ALT, AST, ADH, TC, TG, GSH-Px, SOD, and CYP450 2E1 levels were all increased in the model group (*P* < 0.01), while ALDH and MDA were decreased (*P* < 0.01). Compared with the model group, the detection indicators in the different dose CAG treatment groups could be reversed, and there was a certain dose-effect relationship, that is, the reversal effect of the high and med-dose groups was better (*P* < 0.01). Liver histological observation showed that CAG could alleviate the infiltration of inflammatory cells.

**Conclusion:**

These indicated that CAG had no acute toxicity and exhibited a large safety range, and was first identified to protect against hepatotoxicity through anti-oxidative stress and anti-inflammation, providing a scientific basis for further research into its clinical applications.

## 1 Introduction

Alcoholic liver disease (ALD) is one of the most serious and irreversible form of liver damage associated with alcohol consumption and is the leading cause of liver-related morbidity and mortality worldwide (Qi et al., 2022; Dukić et al., 2023). ALD imposes a substantial global public health burden and is often associated with high rates of hospitalization and mortality, especially in individuals with long-term alcohol abuse (Åberg et al., 2024). Despite intensive research efforts, effective clinical interventions for ALD are still limited, making it an urgent target for therapeutic innovation.

The pathogenesis of ALD is complex and multifactorial. While no single pathway fully explains alcohol-induced liver injury (Obad et al., 2018; Wang et al., 2021), oxidative stress has been identified as a central contributor, particularly in the early stages of the disease progression (Shuai et al., 2021). The liver, responsible for metabolizing approximately 90% of ingested ethanol, relies primarily on the alcohol dehydrogenase (ADH) and aldehyde dehydrogenase (ALDH) pathways. Ethanol is first oxidized by ADH into acetaldehyde and subsequently converted to acetic acid by ALDH, ultimately yielding CO_2_ and H_2_O (Osna et al., 2022; Contreras-Zentella et al., 2022). However, when large amounts of alcohol are consumed over a long period of time, the high concentration of ethanol in the blood activates the microsomal ethanol oxidizing system (MEOS), notably increasing cytochrome P450 2E1 (CYP2E1) activity. This pathway produces a large amount of reactive oxygen species (ROS) and the toxin acetaldehyde during the metabolism of ethanol, causing severe damage to the liver (Liu et al., 2025). In addition to oxidative stress, inflammation plays a key role in the pathogenesis of ALD. Chronic alcohol consumption triggers the release of pro-inflammatory cytokines, including tumour necrosis factor-α (TNF-α), interleukin-6 (IL-6), and interleukin-1β (IL-1β). These cytokines contribute to liver injury by activating immune cells and further enhancing oxidative stress. TNF-α, for example, has been shown to induce hepatocyte apoptosis and promote inflammatory responses, while IL-6 and IL-1β mediate the activation of NF-κB signal, which exacerbates liver inflammation and fibrosis (Zhang X. et al., 2023). These inflammatory pathways, in combination with oxidative stress, create a vicious cycle that accelerates liver damage and progression to cirrhosis. Although synthetic drugs such as tiopronin and bifendatatum have been used to prevent or treat ALD (Nass et al., 2017; Ma et al., 2020), their effectiveness in restoring liver function remains unsatisfactory. Consequently, there is an increasing interest in identifying safer and more effective therapeutic alternatives, particularly from natural or traditional sources.

Traditional Chinese Medicine (TCM) has a long-standing history of preventing and treating alcoholism. Modern medical studies have reported that combinations of TCM and Western medicine may offer enhanced efficacy, particularly in acute alcohol intoxication (Fang J. et al., 2022; Chen Y. et al., 2024). The current development of TCM for relieving alcoholism mainly focuses on two aspects. Firstly, it strengthens the first pass metabolism of ethanol in the gastrointestinal tract, inhibits its gastrointestinal absorption, and reduces the concentration of ethanol in the blood. The second is for the drugs to directly act on the enzyme system involved in ethanol metabolism to improve the elimination rate of ethanol and its metabolites to reduce its damage to liver and other tissues. Several herbal formulas and compounds including Ge Bai Jie Jiu Ye, Song Hua Ge Gen Pian, and Qinggan Huoxue Recipe, have demonstrated efficacy in lowering serum ethanol levels or mitigating alcohol-induced liver injury (Bi et al., 2005; Wang et al., 2014; Lu et al., 2022). For example, LanGui tea, comprising of *Gynostemma pentaphyllum*, *Cinnamomum cassia* and *Ampelopsis grossedentata* may ameliorate acute ALD by activating AMPK and inhibiting NLRP3 signal pathways (Gu et al., 2024). Similarly, acidic polysaccharide from *Schisandra chinensis* may be via inhibiting the expression of CYP2E1 protein and then alleviating oxidative stress injury induced by ethanol (Yuan et al., 2018).

Among these remedies, Chaige anti-alcoholic granules (CAG) represent a modern TCM formula developed based on ancient antidotal prescriptions, clinical expertise, and the TCM principle of internal-external coordination (Hu et al., 2016). Zhang et al. (2018) reviewed historical records and identified 384 single herbs and 429 traditional formulas used for alcoholism. The formula, clinically applied at the Wuhu Hospital of Traditional Chinese Medicine, consists of 13 Chinese medicinal ingredients, including *Bupleuri Radix*, *Puerariae Lobatae Radix*, *Puerariae Lobatae Flos*, *Hoveniae Semen*, *Perillae Caulis*, *Citri Reticulatae Pericarpium*, *Phyllostachydis Henonis Folium*, *Rhei Radix et Rhizoma*, *Plantaginis Herba*, *Vignae Radiatae Testa*, *Amomi Semen*, *Coptidis Rhizoma*, and *Alismatis Rhizoma*. Each medicine in this formula reduces alcohol consumption and protects the liver (Du J et al., 2010; Han et al., 2021; Qu et al., 2022; Zhao et al., 2022). The combination of multiple traditional Chinese medicines often has a synergistic effect. Declinol, a complex containing Kudzu, bitter Herbs (Gentian and Tangerine Peel), and Bupleurum, was found to significantly reduce alcohol use disorder identification test scores of moderate to heavy drinkers in a naturalistic setting. The inclusion of bitters such as Gentian and Tangerine Peel in Declinol provides stimulation of gut TAS2R receptors which is potentially synergistic with the effects of Kudzu (Kushner et al., 2013). The formula was composed of *Dregeasinensis Hemsl* and *Pueraria montana* var. lobata (Willd.) Maesen, can prevent acute alcoholic liver injury (ALI) and repair the intestinal mucosal barrier, the effect is better than Biphenyl bisphenol (Bai et al., 2024). In addition, clinical use discovery that CAG can significantly improve nausea, vomiting, headache, and irritability in patients with acute alcohol intoxication in the non-comatose state and reduce the time of waking up.

Despite promising clinical outcomes, the pharmacological mechanisms and hepatoprotective effects of CAG remain insufficiently characterized. Therefore, this study was conducted to investigate the safety profile and therapeutic potential of CAG in the treatment of ALI. Acute toxicity experiments were firstly conducted in mice, a widely accepted model for preclinical drug safety evaluation (Chen D. H. et al., 2024). Subsequently, an animal model of ALI was established in rats, and the structural changes of liver tissue were observed histologically. The measurement of key biochemical markers, including alanine transaminase (ALT), aspartate transaminase (AST), alcohol dehydrogenase (ADH), Acetaldehyde dehydrogenase (ALDH), triglyceride (TG), total cholesterol (TC), superoxide dismutase (SOD), malondialdehyde (MDA), and glutathione peroxidase (GSH-Px) were performed after the rats were exposed to different CAG treatment conditions. In addition, the protein expression of hepatic cytochrome P450 2E1 (CYP2E1) was determined using Western blotting. The levels of TNF-α, IL-6, and IL-1β were detected in rats using ELISA. Through these investigations, the study aims to elucidate the mechanism of CAG in mitigating alcohol-induced liver injury and to provide a scientific basis for its clinical application in emergency TCM interventions for acute alcoholism.

## 2 Materials and methods

### 2.1 Animals

Male Kunming mice (six-week-old), weighing 18–22 g, were purchased from the SCBS Bio-technology Co. (SCXK 2020-0005, Henan, China). Male Sprague–Dawley (SD) rats (180–220 g) were purchased from Hangzhou Medical College Laboratory Animal Center (SCXK 2019-0002, Zhejiang, China). These animals were maintained under a controlled environment at a room temperature of 25°C ± 1°C and 50% ± 10% humidity on a 12 h light-dark cycle with free access to water and food. The animal experiments were performed in accordance with the Guidelines for Animal Experimentation, with the approval of the Institutional Animal Care and Use Committee of Wannan Medical College (approval number LLSC-2021–192 for mice, approval number LLSC-2022–042 for rats).

### 2.2 Animal treatment

#### 2.2.1 Acute toxicity experiments of CAG

Experiments were conducted after 1 week of acclimatization of the mice to the environment. The experimental animals were randomly divided into two groups (*n* = 10/group). The same volume of distilled water was administered by gavage as the control group. We administered the CAG to the mice for 2 weeks at the clinical dose, without observing any significant adverse reactions. Based on the literature (Wang, 2020; Song, 2023), the concentration of the maximum dosage group (CAG group) was set to the highest concentration at which CAG could dissolve in water to be administrated through the gavage needle, which is 0.78 g mL^-1^, with a maximum volume of 0.4 mL per 10 g of body weight. The mice were administered for 14 days, and their body weights were recorded and their behaviour activities were observed during this period.

#### 2.2.2 CAG hepatoprotective effect

In clinical practice, metadoxine is the main drug used to treat acute alcohol intoxication. It accelerates the elimination of ethanol from the blood, leads to faster recovery from intoxication, and improves behaviour toxic symptomatology (Shpilenya et al., 2002). Based on clinically used doses, 100 mg kg^-1^ of metadoxine was used in the animal studies.

After 1 week of acclimation, the rats were randomly divided into the following groups (*n* = 6): control, model, positive, CAG low-, medium-, high-dose groups. The control and model groups were administered distilled water via gavage, and the other groups were administered metadoxine (100 mg kg^-1^ for the positive group) or CAG (1,575, 3,150, or 6,300 mg kg^-1^ for the low-, medium-, and high-dose groups, respectively) via gavage. A model of ALI was established via the administration of Chinese wine with 56% alcohol (Beijing Red Star Co., Beijing, China) by gavage, as previously reported (Zhang et al., 2023a). Except for the control group, rats were intragastrically administered Chinese wine at a dose of 15 mL kg^-1^ once daily for 14 days. Rats in the control group received distilled water.

#### 2.2.3 Sample collection and liver index determination

The mice and rats were fasted for 12 h at the final stage of the experiment before being anesthetized. The mice were anesthetized with 1.25% ready-to-use tribromoethanol at a dose of 0.2 mL·10 g^-1^. The rats were anesthetized with 2.5% ready-to-use tribromoethanol at a dose of 0.6 mL·100 g^-1^. Blood was collected from the abdominal aorta and centrifuged for 15 min at 3,500 rpm prior to storage at −80°C. The livers of the rats were quickly harvested, rinsed with cold normal saline, and weighed. Parts of the washed livers were fixed in 4% paraformaldehyde (Beijing Lanjieke Technology Co., Beijing, China) for histopathological examination, and the remaining specimens were frozen at −80°C for additional molecular biology analyses.

The organ index was calculated using Equation 1.

$$\text{Organ index }\left(g/g\right)=\text{organ weight }\left(g\right)\;/\text{ body weight }\left(g\right)$$ (1)

### 2.3 Serum biochemical analyses

The ALT, AST, blood urea nitrogen (BUN), creatinine, SOD, GSH-Px, MDA, TC, and TG levels were determined in the serum using the appropriate test kits (Nanjing Jiancheng Bioengineering Institute, Nanjing, Jiangsu, China). In brief, the indicated concentration of rat serum was added into the enzyme-labelled plates and incubated at 37°C for 120 min. Then, the supernatant in the plates was discarded, 100 μL biotinylated antibody was added, and the incubation was continued for 60 min. The plates were washed with the buffer solution thrice. Subsequently, 100 μL streptavidin-HRP working solution was added and incubated at 37°C for 30 min. Following these, the TMB substrate were put into plates and incubated at 37°C for 15 min in dark. Finally, 50 μL stop solution was added, and the OD values were measured by using a microplate reader (1,510, Thermo Fisher Scientific, MA, United States) at 450 nm.

### 2.4 Liver biochemical analyses

Rat liver tissues were homogenized and centrifuged, and the supernatant was collected. The activities of SOD and GSH-Px and the MDA concentration in the liver tissue were determined using assays. Proinflammatory cytokines, including TNF-α, IL-6, and IL-1β, were measured in the liver using ELISA kits (ABclonal Biotechnology Co., Ltd., Wuhan, Hubei, China) according to the manufacturer’s instructions.

### 2.5 Histopathological examination

Liver and kidney tissues were collected from the mice and rats, fixed with 4% paraformaldehyde (Beijing Lanjieke Technology Co., Beijing, China), dehydrated using a graded alcohol series, made transparent using xylene (Wuxi Jingke Chemical Co., Wuxi, Jiangsu, China), and embedded in paraffin (Shanghai Huayong Paraffin Co., Shanghai, China). The paraffinized tissues were cut into 5 μm thick sections, dewaxed using xylene and rehydrated with gradient alcohol. The sections were then stained with hematoxylin (BKMAM Biotechnology Co., Ltd., Changsha, Hunan, China) for 6 min. After differentiation with hydrochloric alcohol, the sections were stained with eosin (BKMAM Biotechnology Co., Ltd.) for 5 min. Thereafter, the stained sections were sealed using neutral balsam (Sinopharm Chemical Reagent Co., Ltd., Shanghai, China) and dried at 37°C for 4 h. Then, the pathological sections were photographed using a light microscope (magnification: ×400) (BX53, Olympus, Tokyo, Japan).

### 2.6 Western blot

Total protein in the liver tissue was extracted from the homogenized liver tissues using ice-cold RIPA buffer (Beyotime Biotechnology, Shanghai, China). SDS-PAGE was performed to separate identical quantities of protein samples, which were then transferred onto PVDF membranes (Millipore, United States). The membranes were blocked with 5% skim milk and incubated overnight at 4°C with the following primary antibodies: rabbit anti-cytochrome P450 2E1 (1:1,000, Abcam, Cambridge, United Kingdom) and GAPDH (37 kDa, 1:5,000, Abcam, Cambridge, United Kingdom). The membranes were respectively incubated with HRP-labeled anti-rabbit IgG (Thermo Fisher Scientific, United States) and HRP-labeled anti-mouse IgG (Thermo Fisher Scientific, United States) for 2 h at 22°C following TBST washing. After incubation with the secondary antibodies, the protein bands were visualized using an ImageQuant™ 800 instrument (Cytiva, Washington D.C., United States), and the protein expression levels were quantified by densitometry using ImageJ software (National Institutes of Health, Bethesda, MD, United States).

### 2.7 Statistical analysis

The data are represented as the mean ± standard deviation. All statistical analyses were performed using SPSS software (version 26.0; IBM Corporation, United States), and images were created using GraphPad Prism 9.0 (GraphPad Software, San Diego, United States). Comparisons between the two groups were performed using Student’s t-test. Multiple groups were compared using a one-way analysis of variance. Statistical significance was set at *P* < 0.05.

## 3 Results

### 3.1 Acute toxicity experiments of CAG

#### 3.1.1 Changes in body weight of mice

**FIGURE 1:**
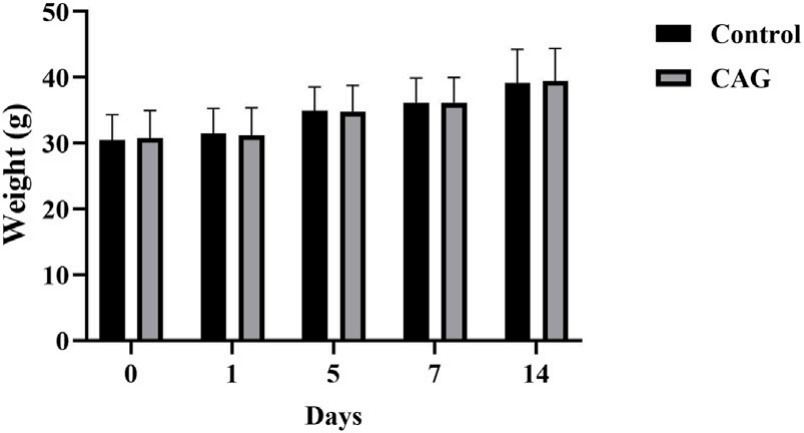
The effects of CAG administration on the body weight of mice in the control group and the CAG group within 14 days (compared the control group, *P* > 0.05, *n* = 10).

As shown in Figure 1, the body weights of mice in both the CAG group and the control group increased slowly within 14 days, and there was no significant difference in body weights between the two groups (*P* > 0.05), which indicated CAG had no significant effect on the growth status of the mice.

#### 3.1.2 Organ index

**FIGURE 2:**
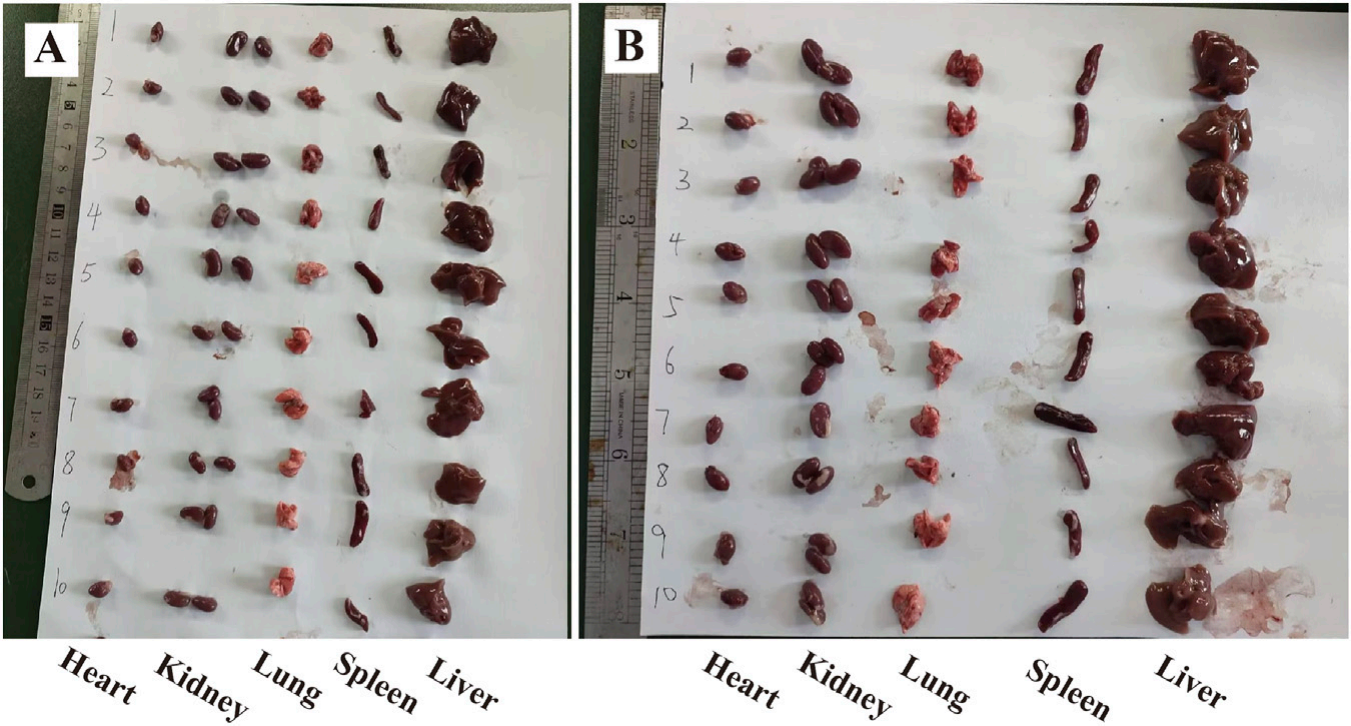
Organs of each group of mice after 14 days of administration of CAG (*n* = 10; **(A)** Organs of control group; **(B)** Organs of the CAG group).

**FIGURE 3:**
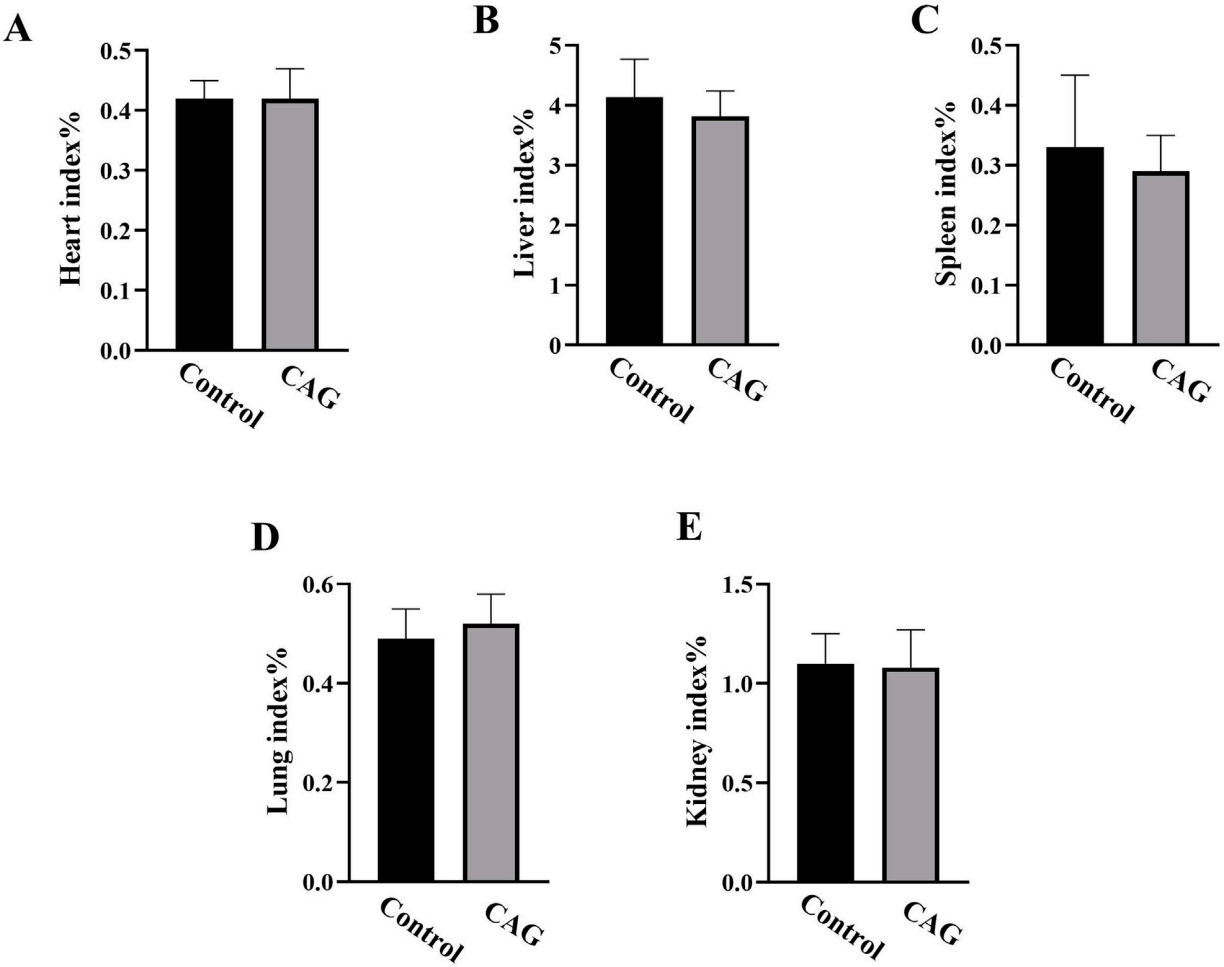
The effect of CAG on the organ index of mice in each group after 14 days of administration (compared with the control group, *P* > 0.05, *n* = 10). (**(A)** Heart index %; **(B)** Liver index %; **(C)** Spleen index %; **(D)** Lung index %; **(E)** Kidney index %).

As shown in Figure 2, the organs in both groups showed normal appearance without congestion. By measurement and calculation, there was no significant difference between both groups about the organ index of heart, liver, spleen, lungs, kidneys, of mice (*P* > 0.05) (Figure 3).

#### 3.1.3 The effect of CAG on blood biochemical parameters in mice

**FIGURE 4:**
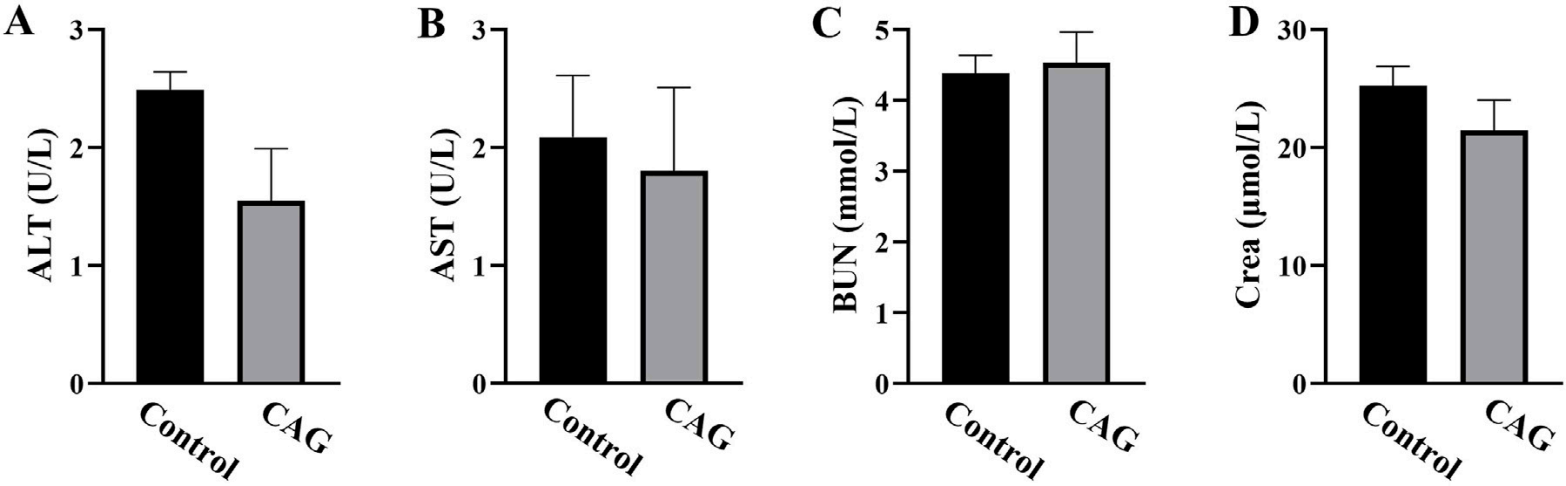
Effects of CAG on blood biochemical indexes of mice after 14 days of administration (compared with the control group, *P* > 0.05, *n* = 10). **(A)** ALT (U/L); **(B)** AST (U/L); **(C)** BUN (mmol/L); **(D)** creatinine (μmol/L).

Serum ALT and AST levels are important markers of hepatocellular damage (Xu et al., 2018), while BUN and creatinine reflect whether kidney function is normal (An et al., 2020; Ding et al., 2021). Figure 4 shows that there was no significant difference in the levels of ALT, AST, BUN and creatinine in the mice between the CAG group and control group (*P* > 0.05). This indicates that CAG had no damage to the liver and kidney functions of the mice.

#### 3.1.4 Liver and kidney histopathology

**FIGURE 5:**
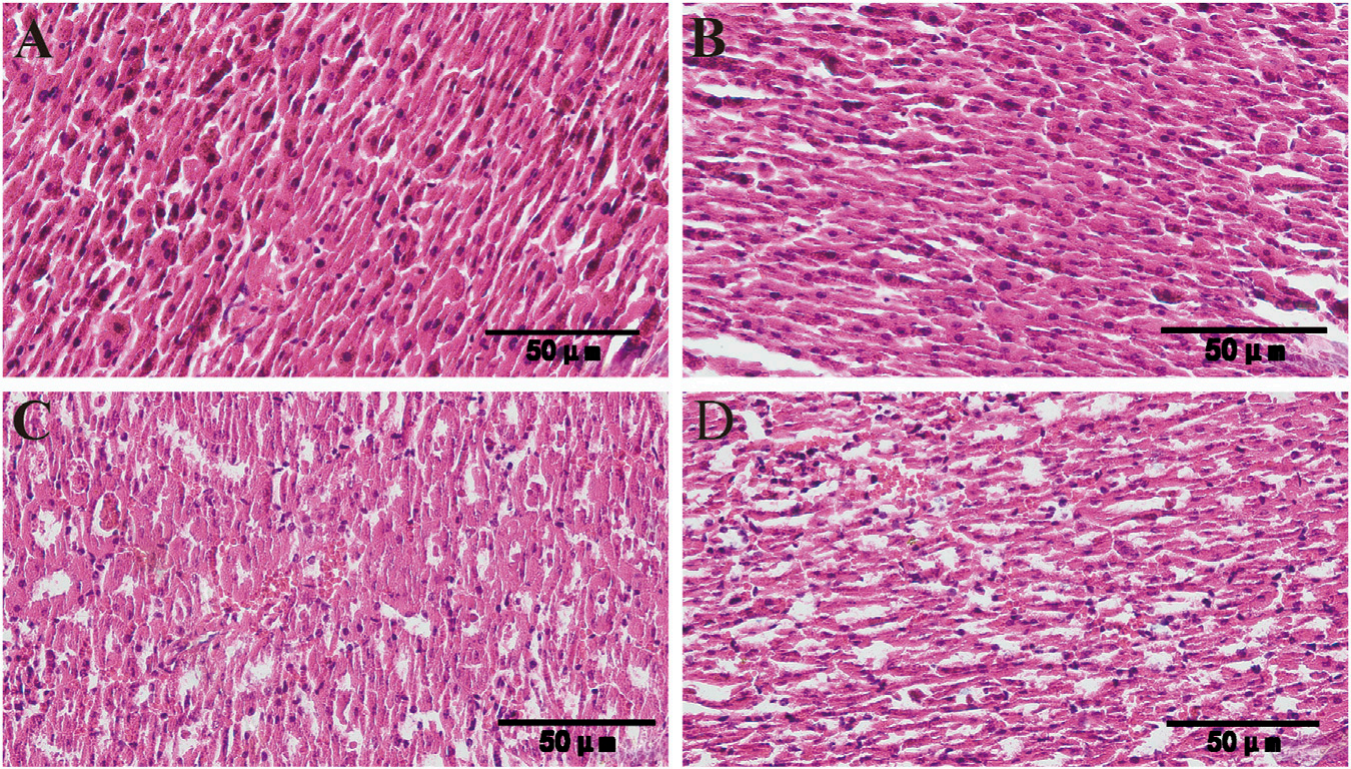
Histopathological changes of liver and kidney of mice after 14 days of administration of CAG (×400) (**(A)** Liver tissue of control group; **(B)** Liver tissue of CAG group; **(C)** Kidney tissue of control group; **(D)** Kidney tissue of CAG group).

Figure 5 HE staining results showed that the hepatic cords of the liver tissues of the control group and the CAG group were arranged in an orderly manner, and no obvious pathological changes were observed. In the renal tissues, the size and structure of glomeruli remained normal, and the renal tubules and collecting ducts were arranged in an orderly manner, and no significant pathological changes.

### 3.2 CAG hepatoprotective effect

#### 3.2.1 Effect of CAG on liver index in rats with ALI

**FIGURE 6:**
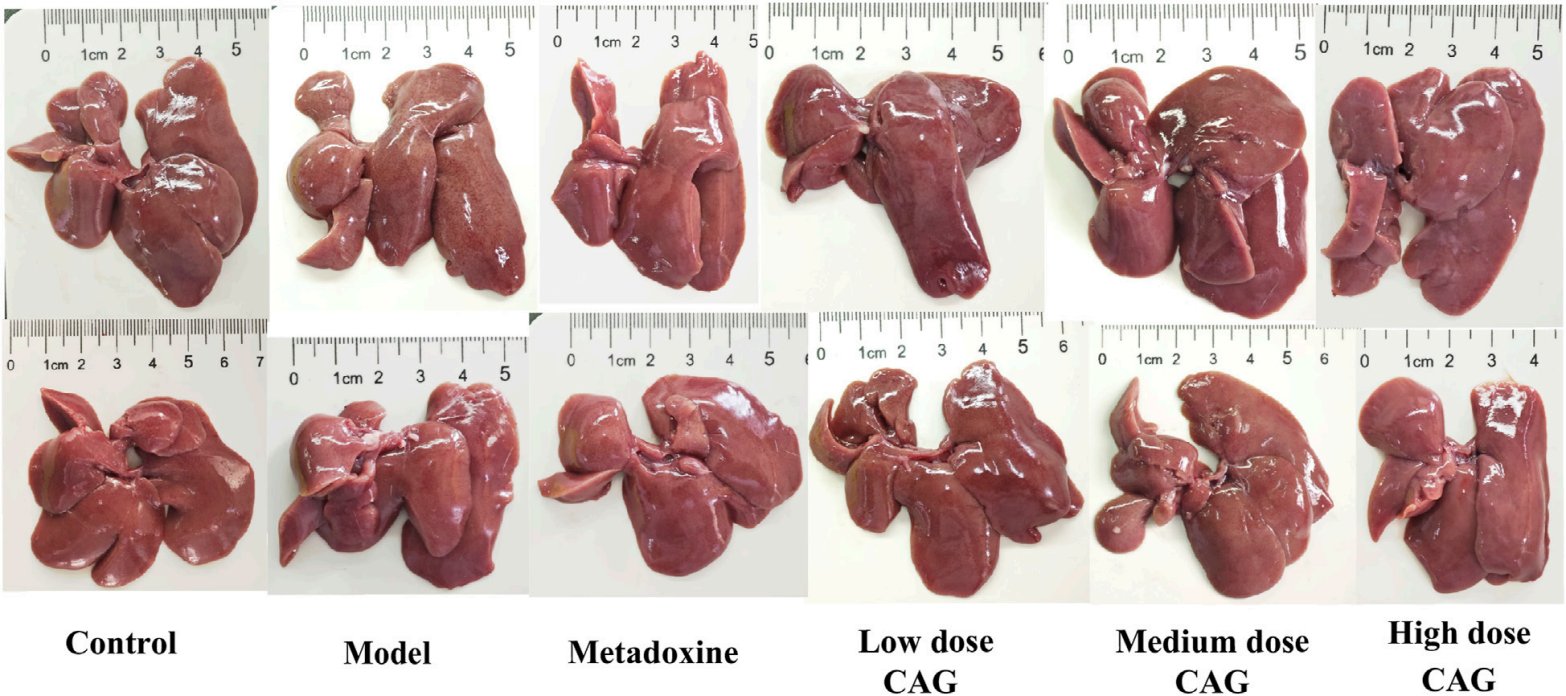
The anatomical diagrams of rat livers.

Excessive alcohol intake resulted in swelling of the liver due to lipid accumulation and inflammatory responses. As depicted in the anatomical diagram of the rat liver in Figure 6, the model group rats showed liver enlargement and a relatively pale appearance compared to the control group, likely due to fat accumulation or bilirubin deposition following liver cell injury. In contrast, no notable liver abnormalities were observed in the metadoxine and CAG group rats.

**FIGURE 7:**
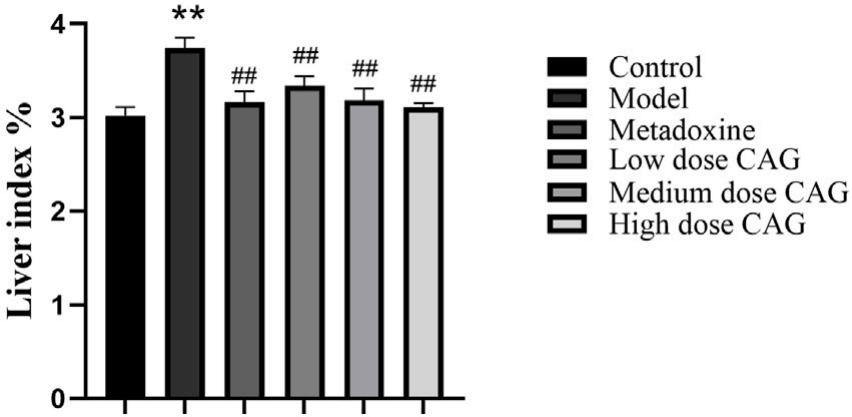
The effect of CAG on liver index in rats with ALI. *n* = 6. ^**^ *P* < 0.01 compared with the control group; ^##^ *P* < 0.01 compared with the model group.

The liver index of the model group was significantly increased compared to that to the control group (*P* < 0.01, Figure 7), verifying that hepatomegaly occurred in the ALI rat model. The high-, medium-, and low-dose groups had significantly lower liver indices than the model group (all *P* < 0.01, Figure 7). The liver index of the high-dose CAG group was lower than that of the metadoxine group (Figure 7), indicating that CAG not only has a protective effect on alcoholic liver injury, but may also have a superior effect than the current drug of choice in clinical settings.

#### 3.2.2 Effect of CAG on ADH and ALDH levels

**FIGURE 8:**
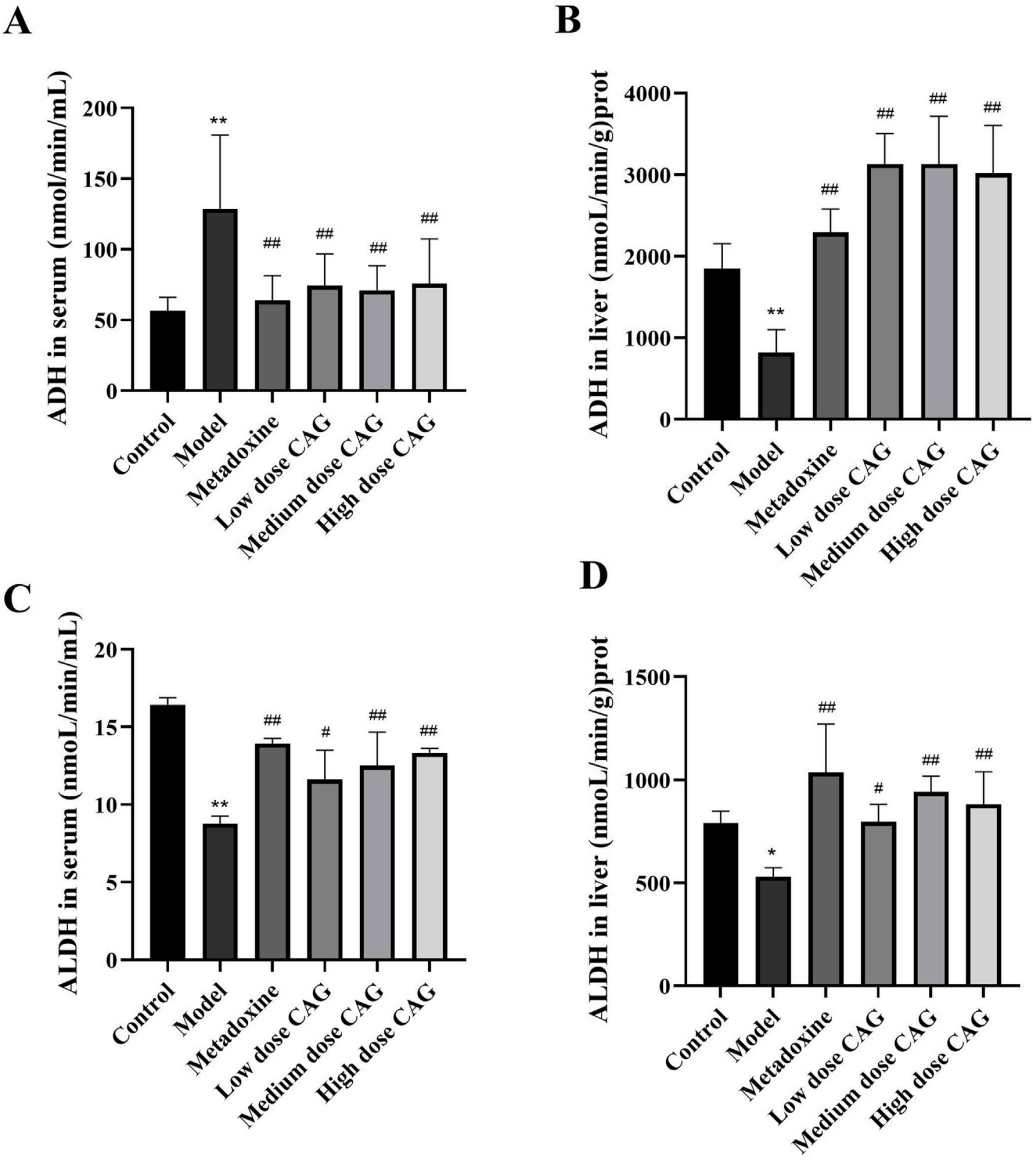
The effect of CAG on the activity of ADH and ALDH in rat serum and liver tissue. **(A)** The content levels of ADH in serum; **(B)** The content levels of ADH in liver; **(C)** The content levels of ALDH in serum; **(D)** The content levels of ALDH in liver). Data were expressed as mean ± SD, n=6. **P < 0.01 compared with the control group; ^##^ *P* < 0.01 compared with the model group.

Alcohol enters the liver through the bloodstream, where it is initially metabolized by ADH into acetaldehyde. Acetaldehyde is then converted into non-toxic acetic acid by aldehyde dehydrogenase ALDH before entering final metabolism. The quantity and activity of these enzymes are innate, so excessive alcohol consumption may require exogenous substances to enhance enzyme activity, accelerate ethanol metabolism, and minimize body damage. As shown in Figure 8, compared to the model group, serum ADH activity in the CAG treatment groups were significantly reduced (*P* < 0.01), while liver ADH activity was significantly increased (*P* < 0.01), suggesting that CAG promote ethanol metabolism by enhancing ADH activity in liver tissue. Additionally, ALDH activity in both serum and liver tissue was significantly increased (*P* < 0.05) in the CAG treatment groups, indicating that the CAG enhances ALDH activity, promote alcohol metabolism, and reduce liver damage. Although metadoxine exhibited slightly greater efficacy compared to CAG, the observed effects of CAG were still notable, indicating its potential as a promising candidate for clinical application.

#### 3.2.3 Effect of CAG on serum enzyme levels

**FIGURE 9:**
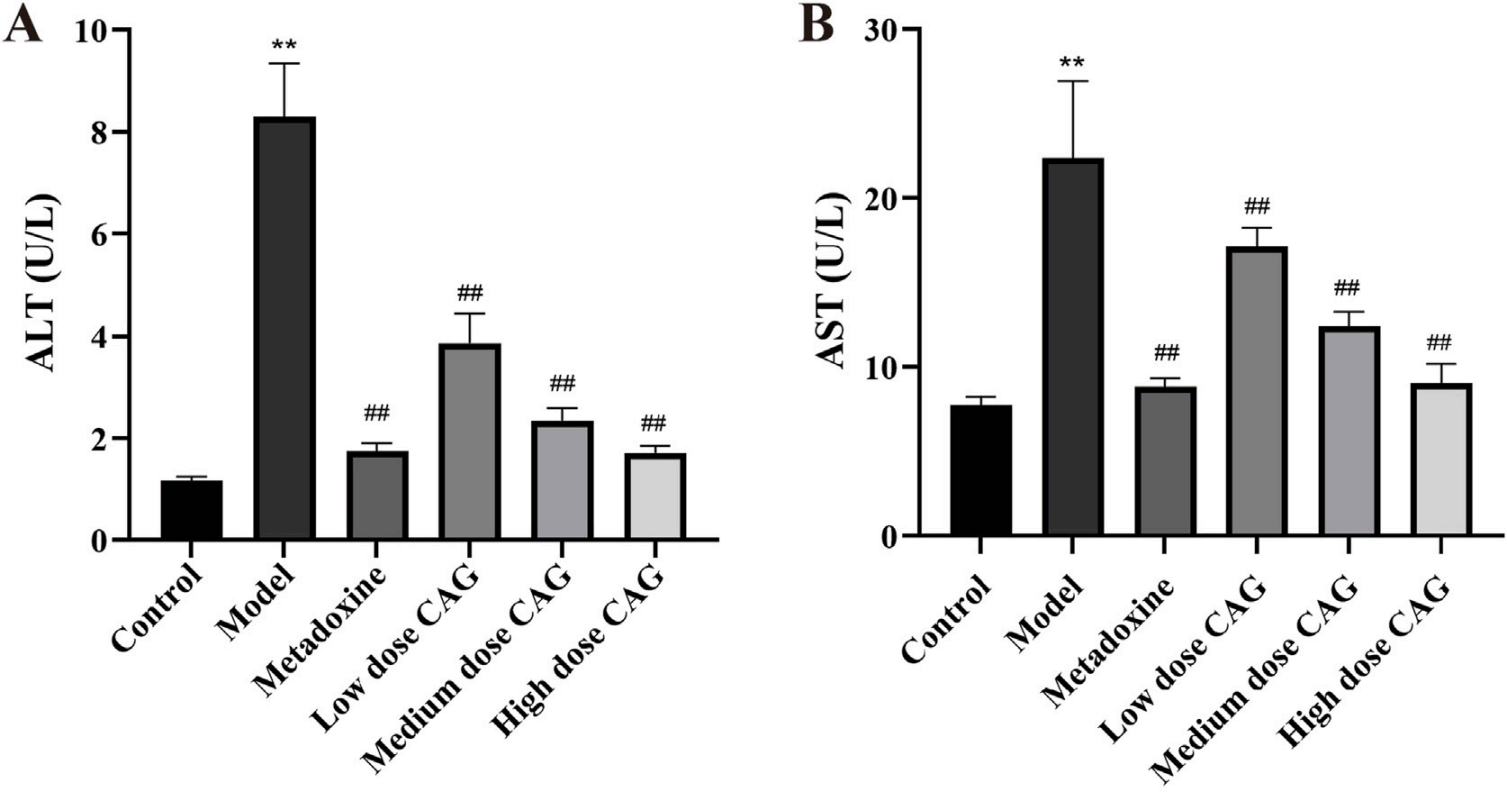
Effects of CAG on ALT and AST in serum of rats with ALI. (**(A)** The content levels of ALT in serum; **(B)** The content levels of AST in serum). Data were expressed as mean ± SD, *n* = 6. ^**^ *P* < 0.01 compared with the control group; ^##^ *P* < 0.01 compared with the model group.

Clinically, AST and ALT are commonly used to reflect liver function. As summarized in Figure 9, serum ALT and AST were significantly higher in the model group than in the control group (*P* < 0.01, *P* < 0.01), indicating the successful establishment of a rat model of liver injury. Serum ALT and AST were decreased in a dose-dependent manner in the CAG treatment groups, suggesting that CAG displays a protective effect against chronic alcoholic liver injury. The high-dose CAG group exhibited effects comparable to those of the metadoxine group. These results suggest that CAG may serve as an effective alternative to metadoxine in the treatment of alcoholic liver disease.

#### 3.2.4 Effect of CAG on serum lipid parameters

**FIGURE 10:**
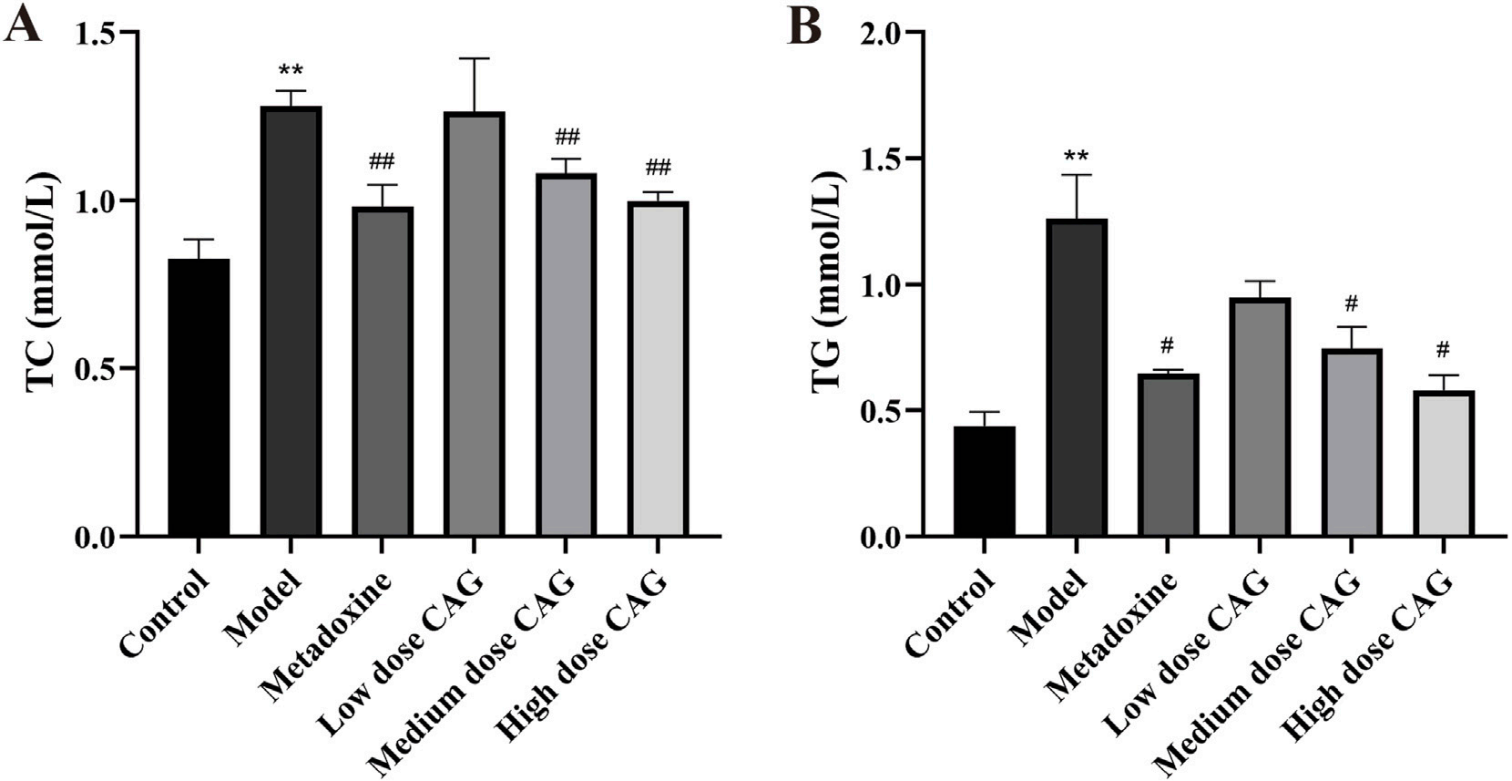
Effects of CAG on TC and TG in rats with ALI. (**(A)**: The content levels of TC in serum; **(B)** The content levels of TG in serum). Data were expressed as mean ± SD, *n* = 6. ^**^ *P* < 0.01 compared with the control group; ^#^ *P* < 0.05, ^##^ *P* < 0.01 compared with the model group.

Lipid metabolism disorder is also a sign of alcohol-induced liver injury in animal models. We detected lipid levels in serum (Figure 10). Serum TC and TG levels were significantly increased in the model group. Serum TC levels were significantly lower in the CAG treatment groups than in the model group (high and medium dose CAG groups, *P* < 0.01). Serum TG levels were significantly lower in the CAG treatment groups than in the model group (high and medium dose CAG groups, *P* < 0.05). TC and TG levels were not significantly different between the low-dose CAG group and the model group. These results suggest that CAG could effectively improve alcohol-induced liver injury in rats. Notably, the high-dose CAG group showed a greater reduction in serum TG levels compared to the metadoxine group, indicating a potentially superior effect in regulating triglyceride metabolism. In contrast, the reduction in TC levels in the high-dose CAG group was comparable to that observed in the metadoxine group.

#### 3.2.5 Effect of CAG on oxidation indices

**FIGURE 11:**
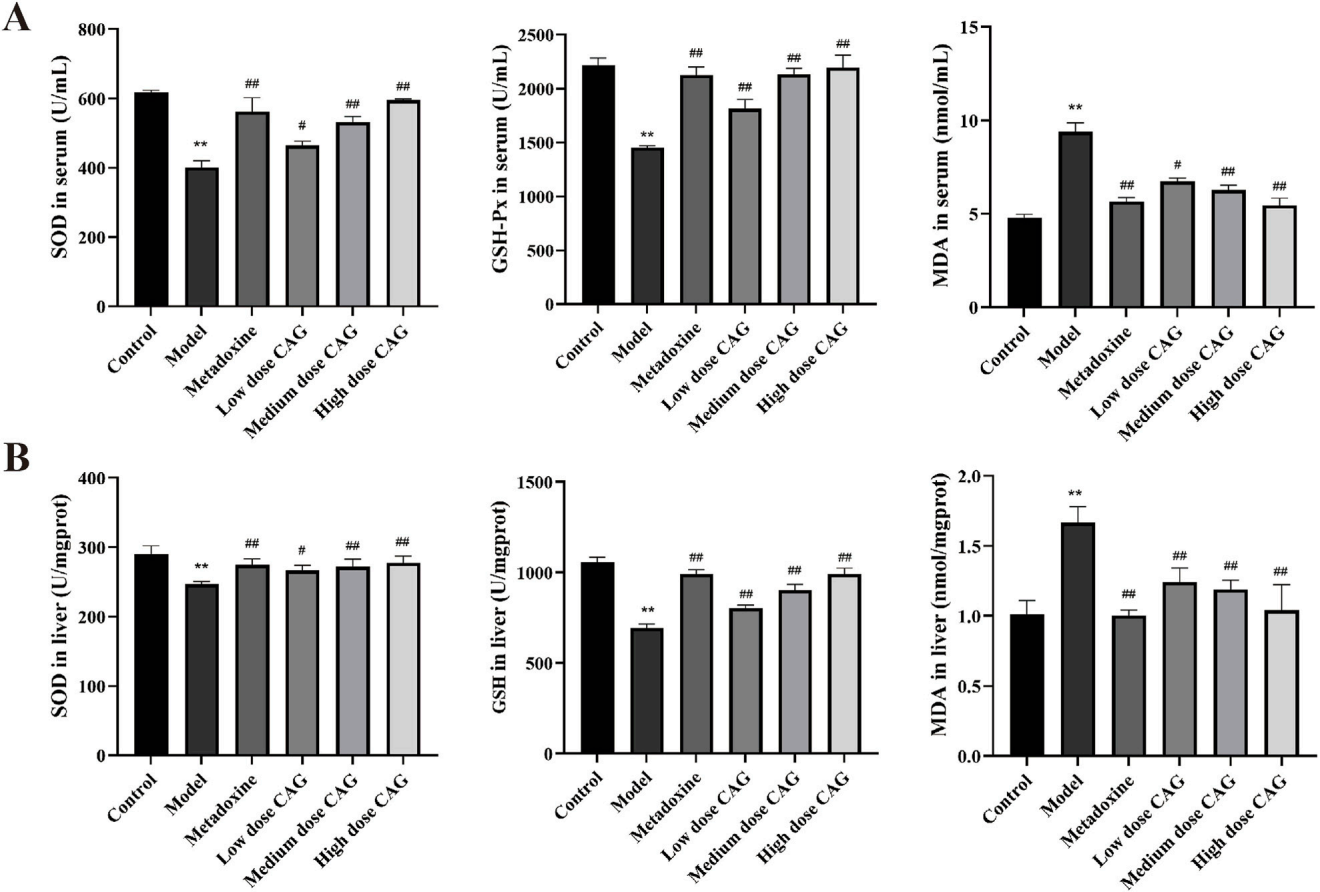
Effects of CAG on the serum and liver oxidation index of rats with ALI. (**(A)**: The levels of SOD, GSH-Px and MDA in serum; **(B)** The levels of SOD, GSH-Px and MDA in liver homogenate). Data were expressed as mean ± SD, *n* = 6. ^**^ *P* < 0.01 compared with the control group; ^#^ *P* < 0.05, ^##^ *P* < 0.01 compared with the model group.

Alcohol induced liver injury can disrupt the homeostasis of the oxidative system in the body. In this study, serum and hepatic MDA levels were significantly higher in the model group than in the control group (all *P* < 0.01). The activities of SOD and GSH-Px were significantly reduced in the model group compared to those in the control group (all *P* < 0.01). However, the activities of SOD and GSH-Px were significantly higher in the high-, medium-, and low-dose CAG groups than in the model group (*P* < 0.01, *P* < 0.05). Serum and liver MDA levels decreased in a dose-dependent manner in the high-, medium-, and low-dose CAG groups (Figure 11). Interestingly, the high-dose CAG group demonstrated superior efficacy in enhancing antioxidant enzyme activities and reducing MDA levels compared to the metadoxine group. These findings suggest that CAG may provide stronger antioxidant protection against alcohol-induced oxidative stress than the currently used positive control.

#### 3.2.6 Effects of CAG on liver levels of TNF-α, IL-6, and IL-1β

**FIGURE 12:**
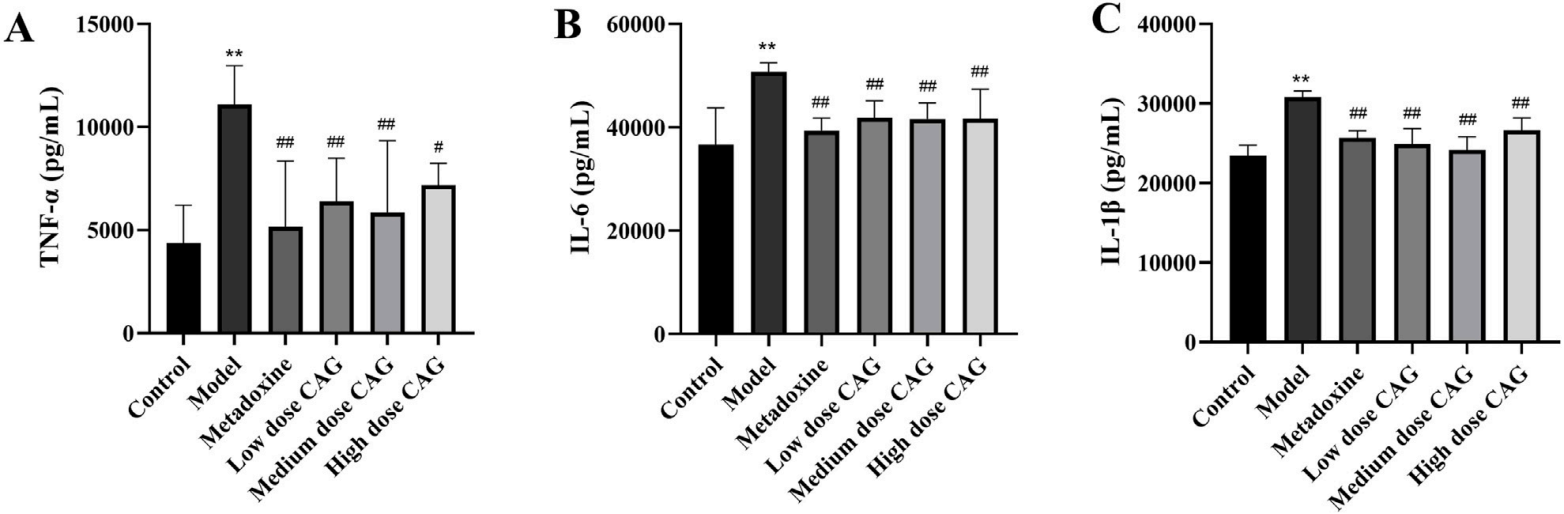
Effects of CAG on liver concentrations of TNF-α, IL-6, and IL-1β in rats with ALI. (**(A)**: The levels of TNF-α in liver; **(B)** The levels of IL-6 in liver; **(C)** The levels of IL-1β in liver). ^**^ *P* < 0.01 compared with the control group; *n* = 6. ^#^ *P* < 0.05, ^##^ *P* < 0.01 compared with the model group.

Alcoholic liver injury may cause inflammation and destruction of liver cells are destroyed (Sun et al., 2020). In addition, necrosis of liver cells may occur (Mehanna et al., 2021). Therefore, the concentrations of inflammatory cytokines in liver were measured. In Figure 12, liver TNF-α, IL-6, and IL-1β were increased significantly in the model group compared to the levels in the control group (all *P* < 0.01). Liver TNF-α, IL-6, and IL-1β were significantly lower in the CAG treatment groups than in the model group (*P* < 0.01, *P* < 0.05). Although the anti-inflammatory effects of the CAG groups were lower than those of the metadoxine group, the results still indicate that CAG exerts a beneficial regulatory effect on inflammatory responses.

#### 3.2.7 Histopathologic changes in the livers of rats with ALI

**FIGURE 13:**
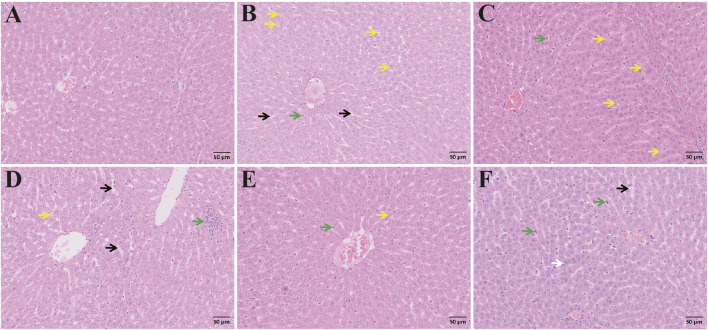
H&E staining of liver sections. Pathological changes of liver tissue from different groups (Note: Black arrow, hepatic sinusoidal dilation; Green arrow, Inflammatory cells; Yellow arrow, Hepatocyte vacuolization; White arrow, Cell proliferation. Magnification: ×200, scale: 50 μm, **(A)** Control group, **(B)** Model group, **(C)** Metadoxine group, **(D)** High dose CAG group, **(E)** Medium dose CAG group, **(F)** Low dose CAG group).

Representative photomicrographs of liver tissues are presented in Figure 13. In the control group, hepatocytes were arranged in double rows, forming liver plates around the central vein. The hepatocytes were irregularly polygonal with large, round nuclei, and adjacent cords were irregularly connected, forming a network of hepatic sinusoids without significant dilation. Slight dilation of hepatic sinusoids was observed. In the model group, hepatic sinusoids showed significant dilation, accompanied by extensive hepatocyte vacuolization and scattered inflammatory cells. In the CAG treatment group, low-dose group showed mild hepatic sinusoidal dilation and inflammatory cell foci, while hepatocyte vacuolization decreased in the medium-high group. Hepatocyte increase was observed in the high-dose group, indicating a post-injury repair response. In the metadoxine group, hepatocyte vacuolization and diffuse distribution of inflammatory cells in the hepatic interstitium were still evident. Compared to the metadoxine group, the high-dose CAG group showed less vacuolization and more organized hepatic architecture, indicating a potentially greater capacity for hepatic tissue repair and inflammation attenuation.

#### 3.2.8 Histopathologic changes in the other tissues of rats with ALI

**FIGURE 14:**
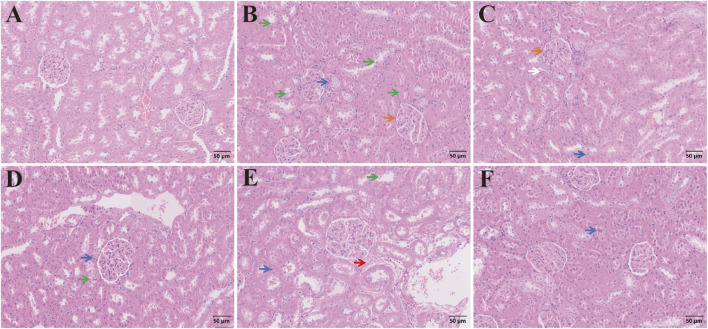
H&E staining of kidney sections. Pathological changes of kidney tissue from different groups (Note: Green arrow, cell necrosis; Blue arrow: inflammatory cells; Orange arrow: glomerulosclerosis; Red arrow: mesenchymal fiber exudation; White arrow: proliferation of tubular epithelial cells. Magnification: ×200, scale: 50 μm, **(A)** Control group, **(B)** Model group, **(C)** Metadoxine group, **(D)** Low dose CAG group, **(E)** Medium dose CAG group, **(F)** High dose CAG group).

Alcohol not only damages the liver but also induces harm to other organs through mechanisms such as inflammation, metabolic dysregulation, and oxidative stress. Therefore, we examined tissue alterations in the kidneys, spleen, lungs, and heart using H&E staining. In Figure 14, the renal tubular epithelial cells in the control group were round, full, and arranged in a neat and regular pattern, with no obvious abnormalities observed in the medulla. There was no noticeable proliferation in the mesenchyme, and no significant inflammatory changes were present. In the model group, tubular epithelial cell necrosis, diffuse infiltration of inflammatory cells into the tubular mesenchyme, and sclerotic glomeruli were observed. In the CAG group, necrosis of tubular epithelial cells was noted, with a few inflammatory cells scattered throughout the mesenchyme. In the medium-dose group, mild fibrous exudation was detected in the mesenchyme, while the high-dose group exhibited only a small number of inflammatory cells, along with the presence of neonatal epithelial cells at the base of necrotic and detached tubular epithelial cells. Compared to the metadoxine group, the CAG group exhibited less severe tubular damage, with a lower degree of inflammatory cell infiltration and fewer proliferating neonatal epithelial cells. The medium and high-dose groups demonstrated significantly less renal tissue damage than the metadoxine group, particularly in terms of reduced inflammatory response and enhanced tubular repair.

**FIGURE 15:**
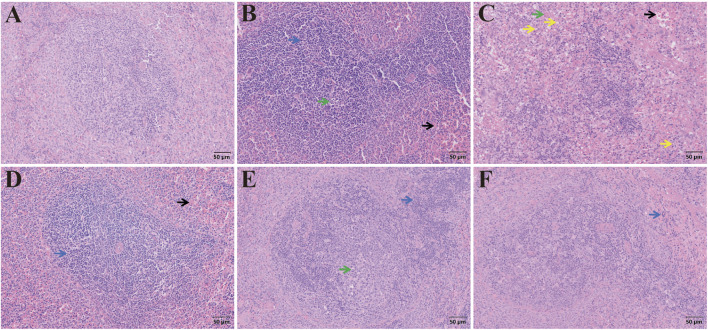
H&E staining of spleen sections. Pathological changes of spleen tissue from different groups (Note: Blue arrow, inflammatory cells; Black arrow, erythrocytes in the splenic sinus; Green arrow: cell necrosis; Yellow arrow: cellular vacuolation. Magnification: ×200, scale: 50 μm, **(A)** Control group, **(B)** Model group, **(C)** Metadoxine group, **(D)** Low dose CAG group, **(E)** Medium dose CAG group, **(F)** High dose CAG group).

There was no obvious abnormality in the spleen tissue of the control group (Figure 15). In the model group, infiltration of inflammatory cells was noted in the spleen, with necrotic cells present in the white pulp and a substantial accumulation of erythrocytes in the splenic sinusoids. The low-dose CAG group showed an increased presence of inflammatory cells in the spleen, along with a marked concentration of erythrocytes in the splenic sinusoids. In the medium- and high-dose groups, the distribution of inflammatory cells gradually diminished, and the number of erythrocytes in the splenic sinusoids was reduced compared to both the model and low-dose CAG groups. In the metadoxine group, extensive cytoplasmic vacuolization was observed, accompanied by a decrease in lymphocytes within the spleen and the presence of necrotic cells. Compared to the metadoxine group, the CAG groups exhibited fewer vacuolated cells and a relatively lesser degree of lymphocyte depletion, indicating a less severe effect on splenic tissue.

**FIGURE 16:**
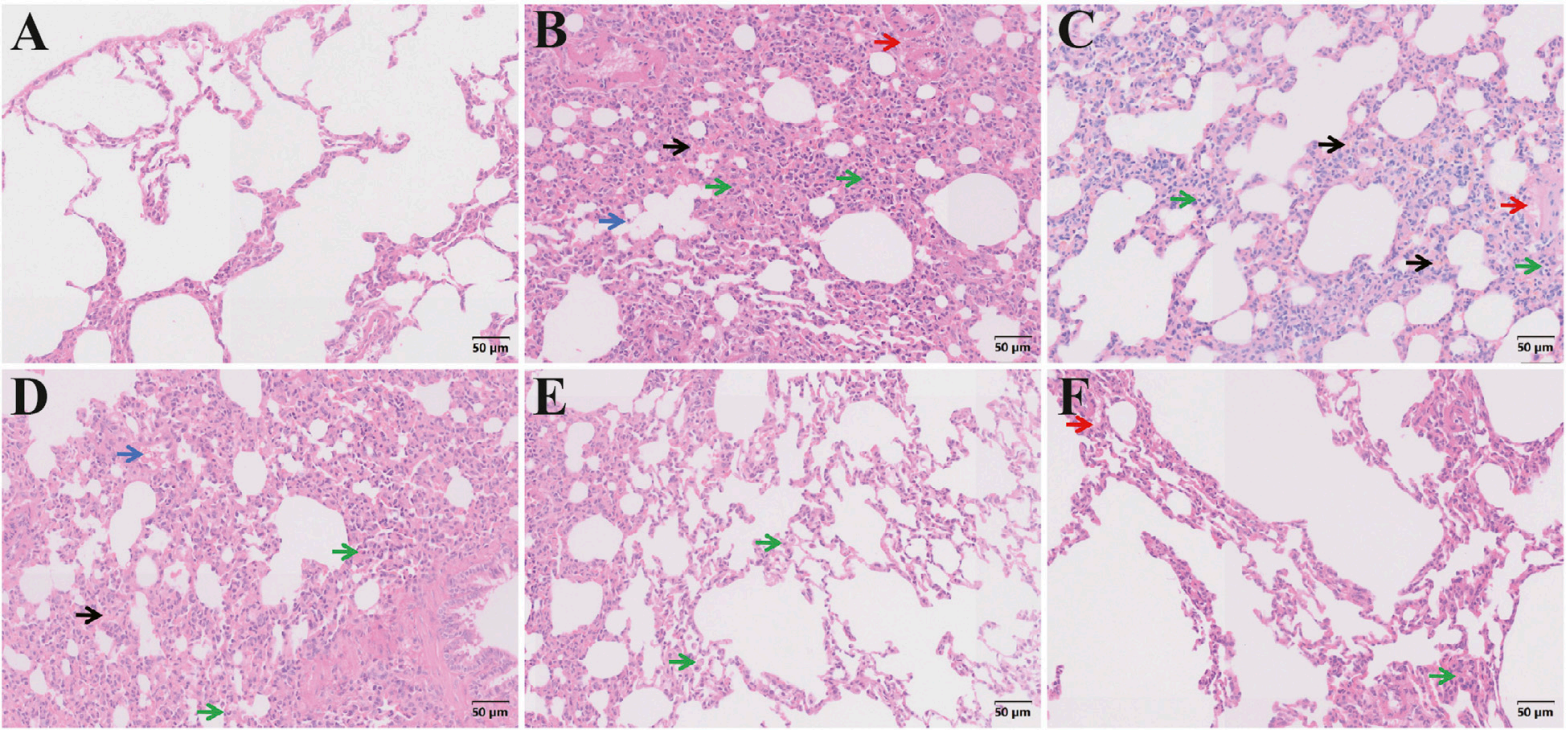
H&E staining of lung sections. Pathological changes of lung tissue from different groups (Note: Green arrow: inflammatory cells; Red arrow: interstitial fiber exudation; Black arrow: interstitial vasodilation; Blue arrow: luminal cell fragments. Magnification: ×200, scale: 50 μm, **(A)** Control group, **(B)** Model group, **(C)** Metadoxine group, **(D)** Low dose CAG group, **(E)** Medium dose CAG group, **(F)** High dose CAG group).

Figure 16 showed that no significant abnormalities or inflammatory changes were observed in the normal lung tissue. In the model group, there was thickening of the pulmonary interstitium, dilation of the pulmonary vessels, fibrous exudation within the interstitial space, and infiltration of interstitial inflammatory cells. These changes were ameliorated following treatment and the CAG group exhibited less severe lung interstitial damage, fewer inflammatory cell infiltrations, and lighter fibrous exudation, indicating a better protective effect than the metadoxine group.

**FIGURE 17:**
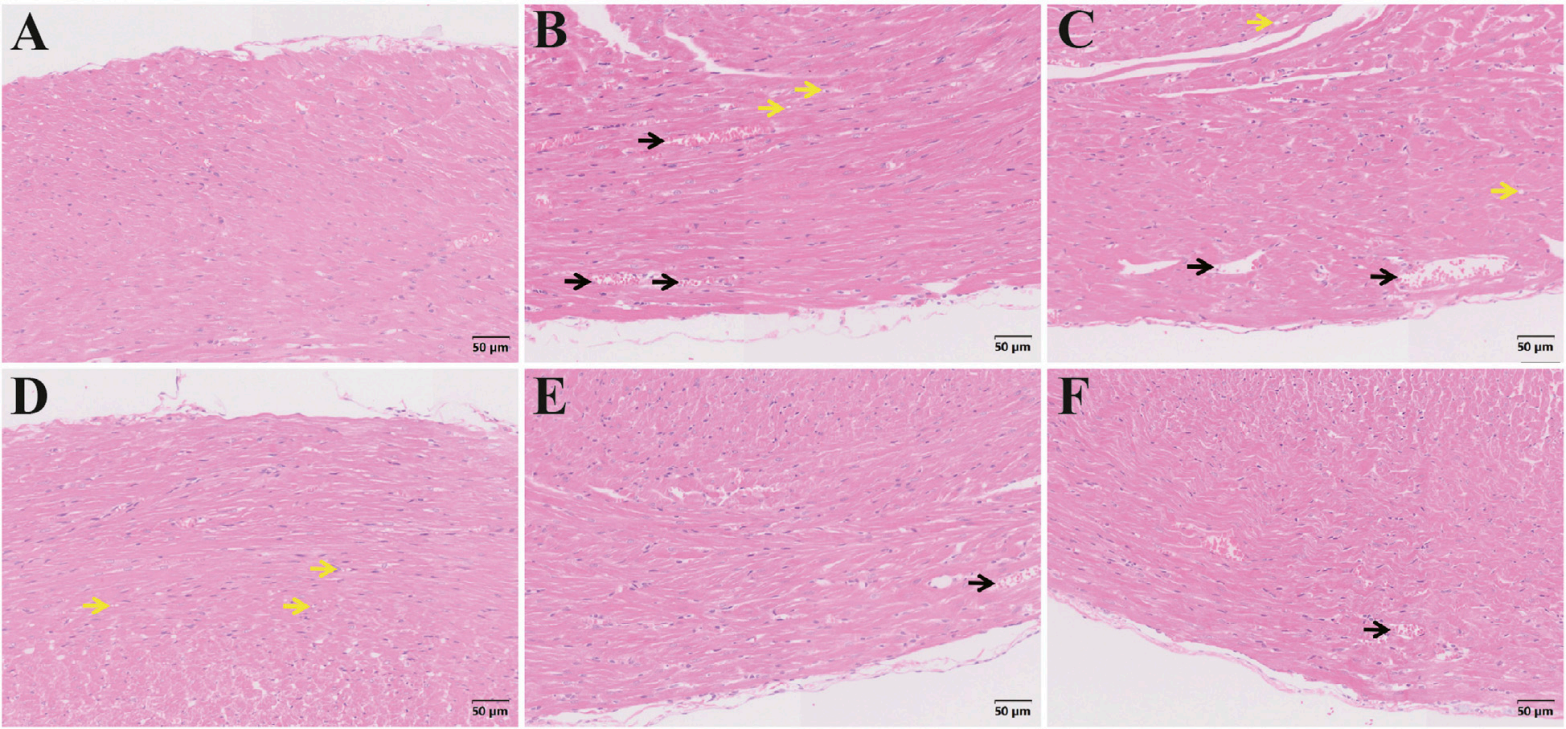
H&E staining of lung sections. Pathological changes of lung tissue from different groups (Note: Black arrow: blood vessel dilation; Yellow arrow: cellular vacuolation. Magnification: ×200, scale: 50 μm, **(A)** Control group, **(B)** Model group, **(C)** Metadoxine group, **(D)** Low dose CAG group, **(E)** Medium dose CAG group, **(F)** High dose CAG group).

As shown in Figure 17, no significant differences were observed in cardiac tissue among the groups. Compared to the metadoxine group, the CAG groups exhibited less severe cardiac tissue damage, with lower levels of cell vacuolization and milder vascular dilation, indicating a better protective effect.

#### 3.2.9 Effects of CAG on the activation of cytochrome P450 2E1 (CYP2E1)

**FIGURE 18:**
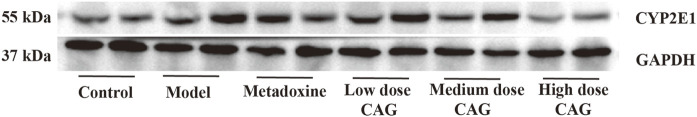
Effects of CAG on the expression of CYP2E1.

Cytochrome P450 2E1 is an ethanol-inducible enzyme, and as the amount and activity of the enzyme increase, ethanol is converted to acetaldehyde, while oxygen free radicals are produced, inducing oxidative stress, and contributing to the formation of alcoholic liver disease. Therefore, it is believed that the higher the activity of CYP2E1 in liver tissue, the greater the liver tissue damage (Jiang et al., 2020). In this study, compared with the control group, the expression of CYP2E1 in the model group increased. The expression of CYP2E1 in the CAG treatment group decreased (Figure 18).

## 4 Discussion

In the preliminary toxicological evaluation, mice were continuously administered twice the clinical dosage of CAG for 14 days, during which no abnormal clinical signs or mortality were observed. To further assess the safety profile of CAG, this study adopts the maximum dosage experiment to evaluate the toxicity of CAG. The results demonstrated that even at the maximum dose administered over 14 days, no significant changes were observed in organ morphology, liver indices, serum biochemical parameters, or histopathological examinations. These findings suggest that CAG exhibits a favorable safety profile and provides a scientific basis for its clinical application. The hepatoprotective effects of CAG were investigated in a rat model of ALI. The model of ALI was established via the administration of wine by gavage (Lamas-Paz et al., 2018), which closely mimics human drinking behavior and causes stable and reproducible liver damage, making it more physiologically relevant than intraperitoneal injection (Fang C. et al., 2022; Brandon-Warner et al., 2012). Following CAG administration, we observed improvements in biochemical markers, liver histopathology, and other organ tissues, supporting its protective role.

Alcohol-induced hepatic injury is typically associated with an elevated liver index (Bai et al., 2020). In this experiment, the liver indices of the high-, medium-, and low-dose CAG groups were significantly lower than that of the model group, indicating that CAG may alleviate hepatic enlargement and swelling. ALT and AST are widely recognized biomarkers for evaluating liver injury, with elevated levels indicative of hepatocellular damage and necrosis (Zhang et al., 2023b; Fang L. et al., 2022). Treatment with CAG significantly reduced AST and ALT levels, further demonstrating its protective effect against hepatocellular injury. Long-term, excessive alcohol consumption may cause an abnormal increase in the hepatic TC and TG levels, resulting in liver metabolic dysfunction (Kashiwagi et al., 2020; Yuan et al., 2020). CAG administration significantly reduced serum TG and TC levels, implying a hepatoprotective effect potentially mediated through the improvement of lipid metabolic disorders. These findings are consistent with previous reports on the hepatoprotective and lipid-lowering properties of *Puerariae Lobatae Radix*, *Puerariae Lobatae Flos*, and *Hoveniae Semen*.

These metabolic improvements suggest that beyond regulating lipid levels, CAG may also exert protective effects by modulating oxidative stress, a key contributor to alcoholic liver injury (Kirpich et al., 2016). Chronic excessive alcohol consumption activates the MEOS, leading to excessive ROS production, which induces lipid peroxidation, elevates MDA levels, and disrupts the balance of antioxidant enzymes such as SOD and GSH-Px, ultimately compromising hepatocyte membrane integrity and resulting in liver cell injury (Yuan et al., 2023; Wang and Mu, 2021; Hua et al., 2023). Although initial alcohol exposure has a major impact on the hepatic redox state due to ethanol metabolism by ADH, repeated exposure to ethanol requires ethanol metabolism via CYP2E1 (Harjumäki et al., 2021). To this end, CYP2E1 has been used as the main index in various animal models of hepatotoxicity, and excessive CYP2E1 expression can increase ROS levels, which may lead to hepatic injury (Lee et al., 2020). CAG exhibited significant antioxidant effects in alcoholic liver injury by reducing MDA levels and enhancing SOD and GSH-Px activities, suggesting its ability to mitigate ROS-induced lipid peroxidation and hepatocellular damage. The observed downregulation of CYP2E1 expression in CAG-treated rats further supports its hepatoprotective mechanism. These findings indicate that CAG may alleviate alcohol-induced oxidative liver damage by enhancing antioxidant defenses and inhibiting CYP2E1-mediated ROS production.

To further elucidate the hepatoprotective mechanisms of CAG, its effects on alcohol-induced inflammation, which is closely linked to oxidative stress, were examined. ROS-induced oxidative stress activates key inflammatory signaling pathways such as the NF-κB and MAPK, leading to the release of pro-inflammatory cytokines (TNF-α, IL-6, and IL-1β), which exacerbate liver damage (Li et al., 2016; Huang et al., 2017; Li et al., 2021). TNF-α, produced by activated macrophages, is an important cytokine in the process of liver inflammation and is a key mediator of liver damage (Jing et al., 2019; Sun et al., 2021). IL-6 activates neutrophils and lymphocytes at the site of inflammation, enhancing self-destructive inflammation (Karatayli et al., 2019; Hu et al., 2021). IL-1β activates macrophages to generate a full range of inflammatory responses including neutrophil infiltration, and its activation promotes liver inflammation (Ambade et al., 2019; Zhou et al., 2021). In this study, treatment with CAG decreased the levels of TNF-α, IL-6, and IL-1β, suppressing inflammatory response and alleviating alcohol-induced liver injury. These results were consistent with the histopathological results. Furthermore, CAG exhibited potential to mitigate alcohol-induced damage in other organs, suggesting a broader protective effect. Histopathological analysis of various organ tissues suggests that, compared with metadoxine, CAG may exert superior protective effects on multiple organs during the progression of ALI. This is consistent with the holistic concept and the multi-target, system-regulating therapeutic characteristics of TCM. Accordingly, CAG may improve multi-organ functions through multiple pathways while exerting anti-inflammatory and antioxidant effects, thus demonstrating a systemic therapeutic advantage over single-target drugs in the treatment of ALI.

Notably, in our previous study, an HPLC method was successfully established to rapidly and efficiently detect six major active components in CAG (Zhao, et al., 2024). Analysis of 20 randomly selected clinical batches demonstrated that the contents of these compounds were consistent, indicating reliable quality control. However, more comprehensive detection methods targeting a broader spectrum of chemical constituents should be developed to ensure the long-term stability and safety of CAG for clinical use.

Despite these encouraging results, this study has several limitations. First, the sample size was relatively small, which may affect the statistical power of the findings. Second, although we evaluated multiple doses of CAG, a clear dose–response relationship was not fully explored. Third, only acute toxicity was assessed, and long-term safety and chronic toxicity studies are still needed. Moreover, pharmacokinetic parameters such as absorption, metabolism, and bioavailability of the active components of CAG were not evaluated and should be investigated in future studies to support its clinical translation.

## 5 Conclusion

CAG demonstrated minimal toxicity in mice and significantly improved the liver function and pathological characteristics in rat model of ALI. The mechanisms were identified to be related to the reduction in oxidative stress, attenuation of alcohol-induced hepatic injury, downregulation of CYP2E1 expression, and inhibition of multiple inflammatory mediators. These findings confirm that CAG effectively protects against the occurrence and development of alcohol-induced liver injury. These results provide a strong foundation for its further development in preclinical and clinical studies targeting ALD. However, challenges such as variability in individual responses and the need for optimized treatment protocols remain as limitations for broader clinical application.

## Data Availability

The original contributions presented in the study are included in the article/supplementary material, further inquiries can be directed to the corresponding authors.

## References

[B1] Åberg F. Jiang Z. G. Cortez-Pinto H. Männistö V. (2024). Alcohol-associated liver disease-global epidemiology. Hepatology 80 (6), 1307–1322. 10.1097/HEP.0000000000000899 38640041

[B2] Ambade A. Lowe P. Kodys K. Catalano D. Gyongyosi B. Cho Y. (2019). Pharmacological inhibition of CCR2/5 signaling prevents and reverses alcohol-induced liver damage, steatosis, and inflammation in mice. Hepatology 69 (3), 1105–1121. 10.1002/hep.30249 30179264 PMC6393202

[B3] An X. Zhang Y. Cao Y. Chen J. Qin H. Yang L. (2020). Punicalagin protects diabetic nephropathy by inhibiting pyroptosis based on TXNIP/NLRP3 pathway. Nutrients 12 (5), 1516. 10.3390/nu12051516 32456088 PMC7284711

[B4] Bai C. Liu T. Xu J. Ma X. Huang L. Liu S. (2020). Effect of high calorie diet on intestinal flora in LPS-induced pneumonia rats. Sci. Rep. 10 (1), 1701. 10.1038/s41598-020-58632-0 32015367 PMC6997398

[B5] Bai Y. Liu F. Zheng L. Wan Y. Fan J. Deng J. (2024). Yajieshaba prevents acute alcoholic liver injury and repairs the intestinal mucosal barrier. J. Ethnopharmacol. 318 (Pt A), 116921. 10.1016/j.jep.2023.116921 37490990

[B6] Bi Y. Guo F. Zhang D. (2005). Experimental study on the mechanism of gerber jiejiu liquor preventing and treating acute alcoholism in mice. J. Jiangxi Univ. Trad. Chin. Med. 17 (5), 50–51. Available online at: https://kns.cnki.net/kcms2/article/abstract?v=4OORb77KhuKANzMg40ocWbNMI3btaBVmKWAzfwu7pPyrfUixvSNmwvNMRn_XaUtWwt-t28Zpr7-TnD3LnCQk_FXrFlYkH3NcQHECULpV0Ha8ispfllY11m8S73gLYWARBZbxPgurutUjQjtSGSjeVbmfrfZfe_07&uniplatform=NZKPT.

[B7] Brandon-Warner E. Schrum L. W. Schmidt C. M. Mckillop I. H. (2012). Rodent models of alcoholic liver disease: of mice and men. Alcohol 46 (8), 715–725. 10.1016/j.alcohol.2012.08.004 22960051 PMC3496818

[B8] Chen D. H. Mao P. J. Diao W. J. Li Q. (2024). Toxicity study of compound granules of hedyotis diffusa: acute toxicity and long-term toxicity. J. Ethnopharmacol. 321, 117434. 10.1016/j.jep.2023.117434 37992881

[B9] Chen Y. Zhang Z. Qian Z. Ma R. Luan M. Sun Y. (2024). Sequentially released liposomes enhance anti-liver cancer efficacy of tetrandrine and celastrol-loaded coix seed oil. Oil. Int. J. Nanomed. 19, 727–742. 10.2147/IJN.S446895 PMC10822770 38288265

[B10] Contreras-Zentella M. L. Villalobos-García D. Hernández-Muñoz R. (2022). Ethanol metabolism in the liver, the induction of oxidant stress, and the antioxidant defense system. Antioxidants 11 (7), 1258. 10.3390/antiox11071258 35883749 PMC9312216

[B11] Ding M. Tang Z. Liu W. Shao T. Yuan P. Chen K. (2021). Burdock fructooligosaccharide attenuates high glucose-induced apoptosis and oxidative stress injury in renal tubular epithelial cells. Front. Pharmacol. 12, 784187. 10.3389/fphar.2021.784187 34955856 PMC8695902

[B12] Du J. He D. Sun L. N. Han T. Zhang H. Qin L. P. (2010). *Semen hoveniae* extract protects against acute alcohol-induced liver injury in mice. Pharm. Biol. 48 (8), 953–958. 10.3109/13880200903300196 20673184

[B13] Dukić M. Radonjić T. Jovanović I. Zdravković M. Todorović Z. Kraišnik N. (2023). Alcohol, inflammation, and, microbiota in alcoholic liver disease. Int. J. Mol. Sci. 24 (4), 3735. 10.3390/ijms24043735 36835145 PMC9966185

[B14] Fang C. Zhang W. Zhang J. Qu N. Cao Z. Pan J. (2022). Research status of the construction and application of common liver injury animal models. Chin. J. Clin. Pharmacol. 38 (3), 276–280. 10.13699/j.cnki.1001-6821.2022.03.020

[B15] Fang J. Wu Y. Gan C. Ruan S. He X. Wang B. (2022). Jia-ga-song-tang protection against alcoholic liver and intestinal damage. Front. Pharmacol. 13, 981706. 10.3389/fphar.2022.981706 36225559 PMC9549243

[B16] Fang L. Wang H. F. Chen Y. M. Bai R. X. Du S. Y. (2022). Baicalin confers hepatoprotective effect against alcohol-associated liver disease by upregulating microRNA-205. Int. Immunopharmacol. 107, 108553. 10.1016/j.intimp.2022.108553 35358777

[B17] Gu M. Chen Y. J. Feng Y. R. Tang Z. P. (2024). LanGui tea, an herbal medicine formula, protects against binge alcohol-induced acute liver injury by activating AMPK-NLRP3 signaling. Chin. Med. 19 (1), 41. 10.1186/s13020-024-00906-0 38439080 PMC10910869

[B18] Han Q. Chen K. Su C. Liu X. Luo X. (2021). Puerarin loaded PLGA nanoparticles: optimization processes of preparation and anti-alcohol intoxication effects in mice. AAPS PharmSciTech 22 (6), 217. 10.1208/s12249-021-02092-w 34386832

[B19] Harjumäki R. Pridgeon C. S. Ingelman-Sundberg M. (2021). CYP2E1 in alcoholic and non-alcoholic liver injury. Roles of ROS, reactive intermediates and lipid overload. Int. J. Mol. Sci. 22 (15), 8221. 10.3390/ijms22158221 34360999 PMC8348366

[B20] Hu G. Zhang R. Huang H. (2016). The latest research progress of traditional Chinese medicine treatment of alcoholism. J. Emerg. Trad. Chin. Med. 25 (1), 110–113. Available online at: https://kns.cnki.net/kcms2/article/abstract?v=4OORb77KhuICa1nmwaHXNfmMGo_H4SolxpLbXChEJCQ9KHrAYHduQXI447rSBdCAFc1EPpn4eh0o0QsP1A-visGcoeeQ1bzOiydnx_eNDojEVylDldLKBPKUzy-ho9shzJW-t_7TedTrwl5dU9uJTxxw6jIo_-W86OotBglGtq4=&uniplatform=NZKPT.

[B21] Hu Y. H. Han J. Wang L. Shi C. Li Y. Olatunji O. J. (2021). α-Mangostin alleviated inflammation in rats with adjuvant-induced arthritis by disrupting adipocytes-mediated metabolism-immune feedback. Front. Pharmacol. 12, 692806. 10.3389/fphar.2021.692806 34305602 PMC8293671

[B22] Hua Z. Hui L. I. Haihua W. (2023). Potential protective effects of the water-soluble Chinese propolis on experimental ulcerative colitis. J. Tradit. Chin. Med. 43 (5), 925–933. 10.19852/j.cnki.jtcm.20230727.002 37679980 PMC10465833

[B23] Huang Q. H. Xu L. Q. Liu Y. H. Wu J. Z. Wu X. Lai X. P. (2017). Polydatin protects rat liver against ethanol-induced injury: involvement of CYP2E1/ROS/Nrf2 and TLR4/NF-κB p65 pathway. Evid.-based Complement. Altern. Med. 2017, 7953850. 10.1155/2017/7953850 PMC5698823 29250126

[B24] Jiang Y. Zhang T. Kusumanchi P. Han S. Yang Z. Liangpunsakul S. (2020). Alcohol metabolizing enzymes, microsomal ethanol oxidizing system, cytochrome P450 2E1, catalase, and aldehyde dehydrogenase in alcohol-associated liver disease. Biomedicines 8 (3), 50. 10.3390/biomedicines8030050 32143280 PMC7148483

[B25] Jing Z. T. Liu W. Xue C. R. Wu S. X. Chen W. N. Lin X. J. (2019). AKT activator SC79 protects hepatocytes from TNF-α-mediated apoptosis and alleviates d-Gal/LPS-induced liver injury. Am. J. Physiol.-Gastroint. Liver Physiol. 316 (3), G387–G396. 10.1152/ajpgi.00350.2018 30629471

[B26] Karatayli E. Hall R. A. Weber S. N. Dooley S. Lammert F. (2019). Effect of alcohol on the interleukin 6-mediated inflammatory response in a new mouse model of acute-on-chronic liver injury. Biochim. Biophys. Acta-Mol. Basis Dis. 1865 (2), 298–307. 10.1016/j.bbadis.2018.11.008 30447270

[B27] Kashiwagi K. Yamaguchi A. Shiba S. Taniki N. Inoue N. Takaishi H. (2020). Moderate alcohol consumption is not associated with subclinical cardiovascular damage but with hepatic fibrosis in non-alcoholic fatty liver disease. Alcohol 89, 1–7. 10.1016/j.alcohol.2020.07.010 32738385

[B28] Kirpich I. A. Miller M. E. Cave M. C. Joshi-Barve S. Mcclain C. J. (2016). Alcoholic liver disease: update on the role of dietary fat. Biomolecules 6 (1), 1. 10.3390/biom6010001 26751488 PMC4808795

[B29] Kushner S. Han D. Oscar-Berman M. William D. B. Madigan M. A. Giordano J. (2013). Declinol, a complex containing kudzu, bitter herbs (gentian, tangerine peel) and *bupleurum*, significantly reduced alcohol use disorders identification test (AUDIT) scores in moderate to heavy drinkers: a pilot study. J. Addict. Res. Ther. 4 (3), 153. 10.4172/2155-6105.1000153 24273684 PMC3835486

[B30] Lamas-Paz A. Hao F. Nelson L. J. Vázquez M. T. Canals S. Gómez D. M. M. (2018). Alcoholic liver disease: utility of animal models. World J. Gastroenterol. 24 (45), 5063–5075. 10.3748/wjg.v24.i45.5063 30568384 PMC6288648

[B31] Lee S. E. Koh H. Joo D. J. Nedumaran B. Jeon H. J. Park C. S. (2020). Induction of SIRT1 by melatonin improves alcohol-mediated oxidative liver injury by disrupting the CRBN-YY1-CYP2E1 signaling pathway. J. Pineal Res. 68 (3), e12638. 10.1111/jpi.12638 32053237

[B32] Li S. Hong Y. Jin X. Li X. Sun E. Zhang G. (2016). Agkistrodon acutus-purified protein C activator protects human umbilical vein endothelial cells against H(2)O(2)-induced apoptosis. Pharm. Biol. 54 (12), 3285–3291. 10.1080/13880209.2016.1224259 27572701

[B33] Li W. Zhao X. Yu T. T. Hao W. Wang G. G. (2021). Knockout of PKC θ gene attenuates oleic acid-induced acute lung injury via reduction of inflammation and oxidative stress. Iran. J. Basic Med. Sci. 24 (7), 986–991. 10.22038/ijbms.2021.56908.12695 34712430 PMC8528254

[B34] Liu T. Zhang F. Feng Y. Han P. Gao Y. (2025). Alcohol-metabolizing enzymes, liver diseases and cancer. Semin. Liver Dis. 45 (1), 99–113. 10.1055/a-2551-3320 40157374 PMC12031026

[B35] Lu Y. Shao M. Xiang H. Wang J. Ji G. Wu T. (2022). Qinggan huoxue recipe alleviates alcoholic liver injury by suppressing endoplasmic reticulum stress through LXR-LPCAT3. Front. Pharmacol. 13, 824185. 10.3389/fphar.2022.824185 35431945 PMC9009225

[B36] Ma Q. G. Wei R. R. Sang Z. P. (2020). Bioactivity-guided isolation of aurone derivatives with hepatoprotective activities from the fruits of Cucumis bisexualis. Z. Naturforsch. C. J. Biosci. 75 (9-10), 327–332. 10.1515/znc-2019-0202 32568735

[B37] Mehanna E. T. Ali A. A. El-Shaarawy F. Mesbah N. M. Abo-Elmatty D. M. Aborehab N. M. (2021). Anti-oxidant and anti-inflammatory effects of lipopolysaccharide from Rhodobacter sphaeroides against ethanol-induced liver and kidney toxicity in experimental rats. Molecules 26 (24), 7437. 10.3390/molecules26247437 34946518 PMC8707101

[B38] Nass F. Schneider B. Wilm S. Kardel B. Gabor E. Merges F. (2017). Influence of tiopronin on the metabolism of alcohol in healthy subjects. Drug Res. 67 (4), 204–210. 10.1055/s-0042-123826 28142160

[B39] Obad A. Peeran A. Little J. I. Haddad G. E. Tarzami S. T. (2018). Alcohol-mediated organ damages: heart and brain. Front. Pharmacol. 9, 81. 10.3389/fphar.2018.00081 29487525 PMC5816804

[B40] Osna N. A. Rasineni K. Ganesan M. Donohue T. J. Kharbanda K. K. (2022). Pathogenesis of alcohol-associated liver disease. J. Clin. Exp. Hepatol. 12 (6), 1492–1513. 10.1016/j.jceh.2022.05.004 36340300 PMC9630031

[B41] Qi X. Zheng S. Ma M. Lian N. Wang H. Chen L. (2022). Curcumol suppresses CCF-mediated hepatocyte senescence through blocking LC3B-Lamin B1 interaction in alcoholic fatty liver disease. Front. Pharmacol. 13, 912825. 10.3389/fphar.2022.912825 35837283 PMC9273900

[B42] Qu J. Chen Q. Wei T. Dou N. Shang D. Yuan D. (2022). Systematic characterization of *puerariae flos* metabolites *in vivo* and assessment of its protective mechanisms against alcoholic liver injury in a rat model. Front. Pharmacol. 13, 915535. 10.3389/fphar.2022.915535 36110520 PMC9468746

[B43] Shpilenya L. S. Muzychenko A. P. Gasbarrini G. Addolorato G. (2002). Metadoxine in acute alcohol intoxication: a double-blind, randomized, placebo-controlled study. *Alcohol*. Clin. Exp. Res. 26 (3), 340–346. 10.1111/j.1530-0277.2002.tb02543.x 11923586

[B44] Shuai C. Xia G. Q. Yuan F. Wang S. Lv X. W. (2021). CD39-mediated ATP-adenosine signalling promotes hepatic stellate cell activation and alcoholic liver disease. Eur. J. Pharmacol. 905, 174198. 10.1016/j.ejphar.2021.174198 34033815

[B45] Song C. (2023). Safety and efficacy evaluation of chaigui granules. Changchun Uni. Chin. Med. 10.26980/d.cnki.gcczc.2023.000589

[B46] Sun S. Wang Y. Du Y. Sun Q. He L. Zhu E. (2020). Oxidative stress-mediated hepatotoxicity in rats induced by ethanol extracts of different parts of *Chloranthus serratus* . Pharm. Biol. 58 (1), 1277–1289. 10.1080/13880209.2020.1859552 33355514 PMC7759245

[B47] Sun S. Zhang J. Li H. Du Y. Li S. Li A. (2021). Anti-inflammatory activity of the water extract of *Chloranthus serratus* roots in LPS-stimulated RAW264.7 cells mediated by the Nrf2/HO-1, MAPK and NF-κB signaling pathways. J. Ethnopharmacol. 271, 113880. 10.1016/j.jep.2021.113880 33508367

[B48] Wang R. Mu J. (2021). Arbutin attenuates ethanol-induced acute hepatic injury by the modulation of oxidative stress and Nrf-2/HO-1 signaling pathway. J. Biochem. Mol. Toxicol. 35 (10), e22872. 10.1002/jbt.22872 34346143

[B49] Wang S. Chen X. Cheng J. Cai T. Wu X. Cheng Z. (2021). Calunduloside E inhibits HepG2 cell proliferation and migration via p38/JNK-HMGB1 signalling axis. J. Pharmacol. Sci. 147 (1), 18–26. 10.1016/j.jphs.2021.05.005 34294368

[B50] Wang S. Hang J. Wang Z. Hang L. Shi L. Li C. (2014). Study dose-effect relationship and mechanisms of hangover effect of Songhua Gegen Tablet. Pharm. Clin. Chin. Mater Med. 30 (4), 110–113. 10.13412/j.cnki.zyyl.2014.04.035

[B51] Wang Z. M. (2020). Clinical study on the treatment of acute alcoholism in non-coma period by shuang ge jie jiu decoction. Anhui Uni. Chin. Med. 10.26922/d.cnki.ganzc.2020.000021

[B52] Xu L. Yu Y. Sang R. Li J. Ge B. Zhang X. (2018). Protective effects of taraxasterol against ethanol-induced liver injury by regulating CYP2E1/Nrf2/HO-1 and NF-κB signaling pathways in mice. Oxidative Med. Cell. Longev. 2018, 8284107. 10.1155/2018/8284107 PMC6174809 30344887

[B53] Yuan H. Duan S. Guan T. Yuan X. Lin J. Hou S. (2020). Vitexin protects against ethanol-induced liver injury through Sirt1/p53 signaling pathway. Eur. J. Pharmacol. 873, 173007. 10.1016/j.ejphar.2020.173007 32045602

[B54] Yuan R. Tao X. Liang S. Pan Y. He L. Sun J. (2018). Protective effect of acidic polysaccharide from Schisandra chinensis on acute ethanol-induced liver injury through reducing CYP2E1-dependent oxidative stress. Biomed. Pharmacother. 99, 537–542. 10.1016/j.biopha.2018.01.079 29902864

[B55] Yuan Y. Wu F. Zhang F. Li X. Wu X. Fu J. (2023). Hepatoenteric protective effect of melanin from Inonotus hispidus on acute alcoholic liver injury in mice. Mol. Nutr. Food Res. 67 (14), e2200562. 10.1002/mnfr.202200562 37162033

[B56] Zhang R. Liu Q. Huang H. (2018). Expert consensus on TCM diagnosis and treatment of acute alcoholism. J. Emerg. Trad. Chin. Med. 27 (10), 1693–1696. Available online at: https://kns.cnki.net/kcms2/article/abstract?v=4OORb77KhuJcwd8Q0N-W8xiB-M7fzX9CFCLsiP5WKqMNAs9AjGpvdK9-W50abJYRKbRuEX1yWUgxXZxgq2BNCoiiYeX0cBBT8chRtvn_zU6JRJFPFxOsl2P9g6TY63eL4OY721mjLoROmCYTRQ2feAxp6lG0X76FqIMzhAOpbdw=&uniplatform=NZKPT.

[B57] Zhang S. Zhang X. Lu S. Luo Y. Sun X. (2023a). Protective effect of naringin on alcohol-induced acute liver injury. Chin. J. Exp. Trad. Med. Form. 29 (01), 61–66. 10.13422/j.cnki.syfjx.20221703

[B58] Zhang S. Zhu P. Yuan J. Cheng K. Xu Q. Chen W. (2023b). Non-alcoholic fatty liver disease combined with rheumatoid arthritis exacerbates liver fibrosis by stimulating co-localization of PTRF and TLR4 in rats. Front. Pharmacol. 14, 1149665. 10.3389/fphar.2023.1149665 37346294 PMC10279862

[B59] Zhang X. Dong Z. Fan H. Yang Q. Yu G. Pan E. (2023). Scutellarin prevents acute alcohol-induced liver injury via inhibiting oxidative stress by regulating the Nrf2/HO-1 pathway and inhibiting inflammation by regulating the AKT, p38 MAPK/NF-κB pathways. J. Zhejiang Univ. Sci. B 24 (7), 617–631. 10.1631/jzus.B2200612 37455138 PMC10350365

[B60] Zhao F. Y. Chen Y. Zhang R. Z. Bi M. F. Gao H. H. Yang X. X. (2024). Simultaneous determination of six active components in chaige Hangover-alleviating granules by HPLC. Trad. Chin. Drug Res. Clin. Pharmacol. 35 (12), 1902–1906. 10.19378/j.issn.1003-9783.2024.12.013

[B61] Zhao W. Peng D. Li W. Chen S. Liu B. Huang P. (2022). Probiotic-fermented *Pueraria lobata* (willd.) ohwi alleviates alcoholic liver injury by enhancing antioxidant defense and modulating gut microbiota. J. Sci. Food. Agric. 102 (15), 6877–6888. 10.1002/jsfa.12049 35655427

[B62] Zhou H. H. Zhang Y. M. Zhang S. P. Xu Q. X. Tian Y. Q. Li P. (2021). Suppression of PTRF alleviates post-infectious irritable bowel syndrome via downregulation of the TLR4 pathway in rats. Front. Pharmacol. 12, 724410. 10.3389/fphar.2021.724410 34690766 PMC8529073

